# Predicting the Potential Climatic Suitability of Typical Poisonous Weeds on the Qinghai–Tibet Plateau Under Climate Change Using Biomod2 and a Single Global Climate Model

**DOI:** 10.3390/plants15203219

**Published:** 2026-10-20

**Authors:** Ruiqi Yang, Hu Ma, Wenwen Guo, Siqing Li, Wenying Wang, Wenlong Li

**Affiliations:** 1 Department of Life Sciences, Qinghai Normal University, Xining 810008, China; 17797080775@163.com; 2 State Key Laboratory of Herbage Improvement and Grassland Agro-Ecosystems, College of Pastoral Agriculture Science and Technology, Lanzhou University, Lanzhou 730030, China; mh980819@163.com (H.M.); xiaowzye@163.com (W.G.); lisq2024@lzu.edu.cn (S.L.)

**Keywords:** Qinghai–Tibet Plateau, poisonous weeds, climate change, ensemble model, species distribution

## Abstract

The expansion of poisonous weeds on the Qinghai–Tibet Plateau (QTP) poses increasing challenges to alpine grassland ecosystems and livestock production. Using the Biomod2 ensemble modelling framework, we assessed the current and future potential climatic suitability for five poisonous weed species—*Stellera chamaejasme*, *Thermopsis lanceolata*, *Gymnaconitum gymnandrum*, *Oxytropis kansuensis*, and *Ligularia virgaurea*—using climatic and topographic predictors, with future projections based on the single global climate model BCC-CSM2-MR under SSP1–2.6, SSP3–7.0, and SSP5–8.5. Precipitation of the warmest quarter (Bio18) and mean temperature of the wettest quarter (Bio8) showed the highest average variable importance across species, although their contributions varied among model realizations. Under current climatic conditions, suitable habitats were concentrated mainly in the eastern and southeastern QTP, with *G. gymnandrum* occupying the largest total and highly suitable areas. Under the BCC-CSM2-MR projections, total suitable habitat increased for all five species across the three SSPs, although the magnitude and spatial pattern of change varied among species, suitability classes, scenarios, and periods. *T. lanceolata* showed the greatest relative increase in suitable habitat, whereas the highly suitable habitat of *O. kansuensis* declined by approximately 94% under SSP5–8.5 in both future periods despite an increase in its total suitable area. These findings provide a basis for ecological risk assessment, targeted monitoring, and adaptive grassland management under future climate change.

## 1. Introduction

The Qinghai–Tibet Plateau (QTP), often referred to as the “Roof of the World” and the “Water Tower of Asia” [1], supports extensive alpine meadow and grassland ecosystems that play critical roles in biodiversity conservation, ecosystem stability, and water resource regulation [2]. As one of the regions most sensitive to global climate change, the QTP has experienced a warming rate significantly exceeding the global average [3]. Under the combined pressures of climate warming and long-term grazing disturbance, grassland degradation has intensified across large areas of the plateau. This process has reduced the competitiveness of palatable forage species, altered plant community composition, and created increasingly favorable climatic conditions for the establishment of poisonous weeds, thereby increasing the extent of climatically suitable habitats for these species [4,5].

The increasing occurrence of poisonous weeds has become an important indicator of grassland degradation and changes in plant community composition in alpine ecosystems. This study selected five representative species—*Stellera chamaejasme* L., *Thermopsis lanceolata* R.Br., *Gymnaconitum gymnandrum* (Maxim.) Wei Wang & Z.D.Chen, *Oxytropis kansuensis* Bunge, and *Ligularia virgaurea* (Maxim.) Mattf. ex Rehder & Kobuski. These species were selected because of their documented relevance to regional grassland ecology and management and the availability of georeferenced occurrence records [6,7,8,9,10,11]. They also encompass contrasting life histories: for example, *G. gymnandrum* is annual, whereas *T. lanceolata* and *O. kansuensis* are perennial. These differences provide ecological context for comparing their projected responses, although life-history traits were not explicitly included as predictors. Taxonomic nomenclature follows Plants of the World Online (POWO), in which *Aconitum gymnandrum* Maxim. is treated as a synonym of *Gymnaconitum gymnandrum*. These species occur naturally on the QTP, although their geographic ranges extend beyond the study area. Their increasing occurrence and dominance in degraded grasslands have been associated with changes in plant community composition, reduced forage quality, and adverse effects on grassland ecosystem functioning and livestock production [12]. Accordingly, these five species provide ecologically relevant examples for comparative modelling and regional risk assessment.

To date, several studies have investigated the potential distributions of poisonous or invasive plant species on the QTP, demonstrating that climatic factors strongly influence habitat suitability and potential distribution patterns [11,13]. Broader multi-species assessments of invasive plants have also been conducted across the QTP [14]. Recent studies have further evaluated future distribution dynamics of multiple native invasive plant species under CMIP6 SSP scenarios [15]. However, comparative assessments focusing specifically on multiple representative poisonous weed species using ensemble SDMs remain limited. Such information can support regional ecological risk assessment, biodiversity conservation, and targeted monitoring and management [16].

To address these knowledge gaps, this study selected five representative poisonous weed species on the QTP and employed the Biomod2 ensemble modelling framework. Although single-algorithm approaches (e.g., MaxEnt) are widely used in species distribution modelling, their predictions may be affected by algorithm-specific biases, overfitting, and structural uncertainties, particularly when projecting future changes in climatic suitability. The Biomod2 framework integrates multiple modelling algorithms, reducing dependence on any single algorithm and allowing examination of among-algorithm variation [17,18,19]. The contribution of this study lies in comparing species-specific changes in total suitable area and suitability-class composition, particularly the potential coexistence of habitat expansion and contraction of highly suitable areas. Specifically, this study addressed the following questions: (1) Which climatic and topographic variables contribute most to predictions of potential suitability for the five species? (2) What are the current potential distribution patterns and highly suitable habitats of these species across the QTP? and (3) How do total suitable area, suitability-class composition, and habitat gains and losses vary among species under different future climate scenarios (SSP1–2.6, SSP3–7.0, and SSP5–8.5)? Because species distribution models estimate potential climatic suitability rather than realized future distributions, the projections presented in this study should be interpreted as potential distribution patterns under an implicit unlimited dispersal assumption. The findings can inform species-specific monitoring and grassland management priorities when combined with field observations.

## 2. Results

### 2.1. Model Uncertainty

Among-algorithm variability, expressed as the spatial mean of pixel-level standard deviations (SDs), ranged from 0.073 to 0.188 across species and climate scenarios. Mean SD values were lower under the current climate than under future climate scenarios and generally increased towards the 2070s.

Among the five poisonous weed species, *Thermopsis lanceolata* consistently exhibited the highest mean SD, increasing from 0.142 under the current climate to 0.188 under SSP3–7.0 during the 2070s. In contrast, *Ligularia virgaurea* showed the lowest mean SD under the current climate (0.073), with values ranging from 0.125 to 0.154 under future climates. *Oxytropis kansuensis* had the lowest or jointly lowest mean SD across future projections, ranging from 0.122 to 0.139. *Gymnaconitum gymnandrum* had a mean SD of 0.087 under the current climate and values ranging from 0.136 to 0.167 under future climates, whereas *Stellera chamaejasme* showed intermediate values across all scenarios.

**Figure 1:**
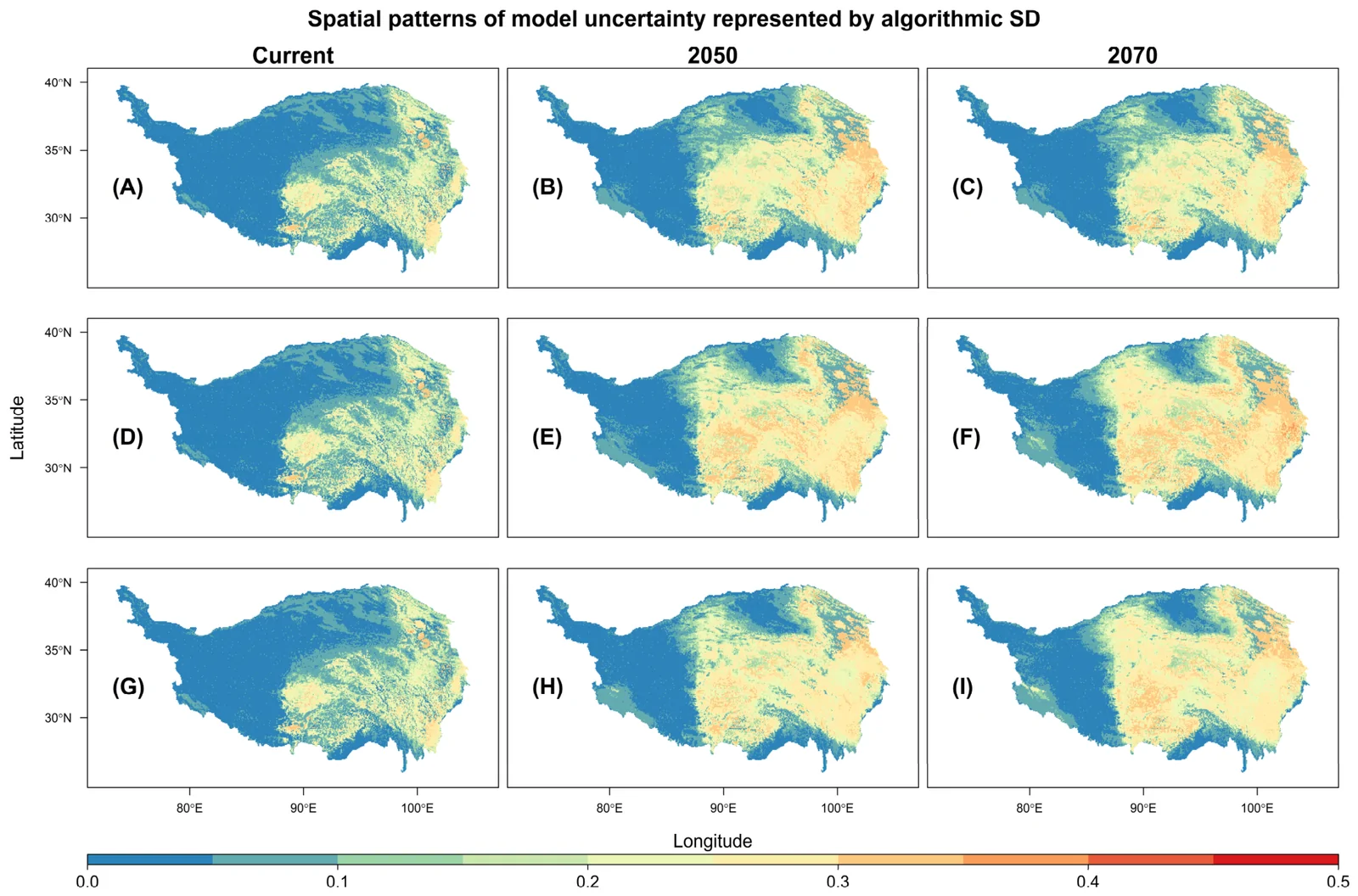
Spatial patterns of model uncertainty for the ensemble species distribution model of *Stellera chamaejasme* under current and future climate scenarios on the Qinghai–Tibet Plateau. (**A**,**D**,**G**) Current; (**B**) SSP1–2.6, 2050s; (**C**) SSP1–2.6, 2070s; (**E**) SSP3–7.0, 2050s; (**F**) SSP3–7.0, 2070s; (**H**) SSP5–8.5, 2050s; and (**I**) SSP5–8.5, 2070s.

**Figure 2:**
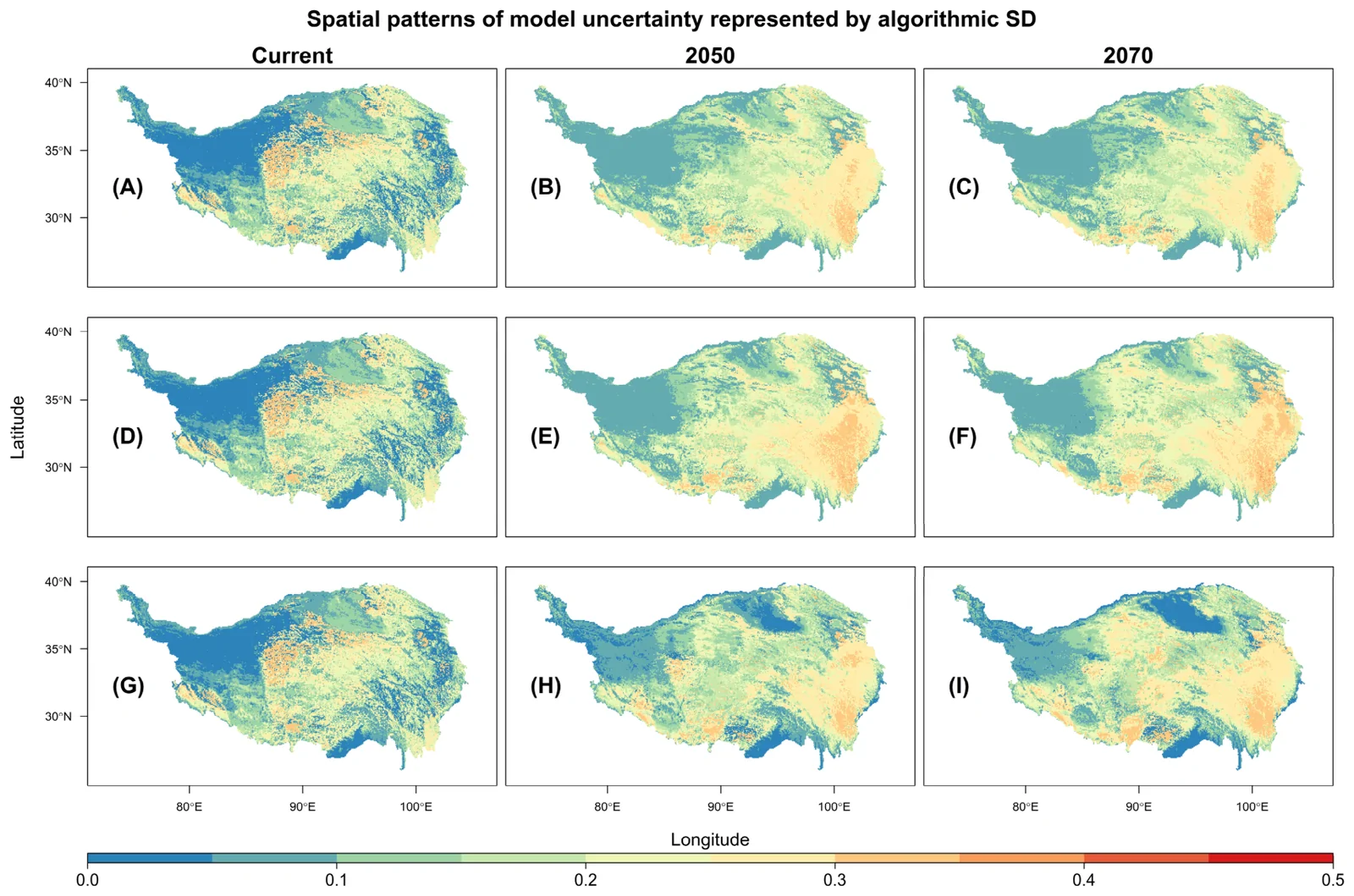
Spatial patterns of model uncertainty for the ensemble species distribution model of *Thermopsis lanceolata* under current and future climate scenarios on the Qinghai–Tibet Plateau. (**A**,**D**,**G**) Current; (**B**) SSP1–2.6, 2050s; (**C**) SSP1–2.6, 2070s; (**E**) SSP3–7.0, 2050s; (**F**) SSP3–7.0, 2070s; (**H**) SSP5–8.5, 2050s; and (**I**) SSP5–8.5, 2070s.

**Figure 3:**
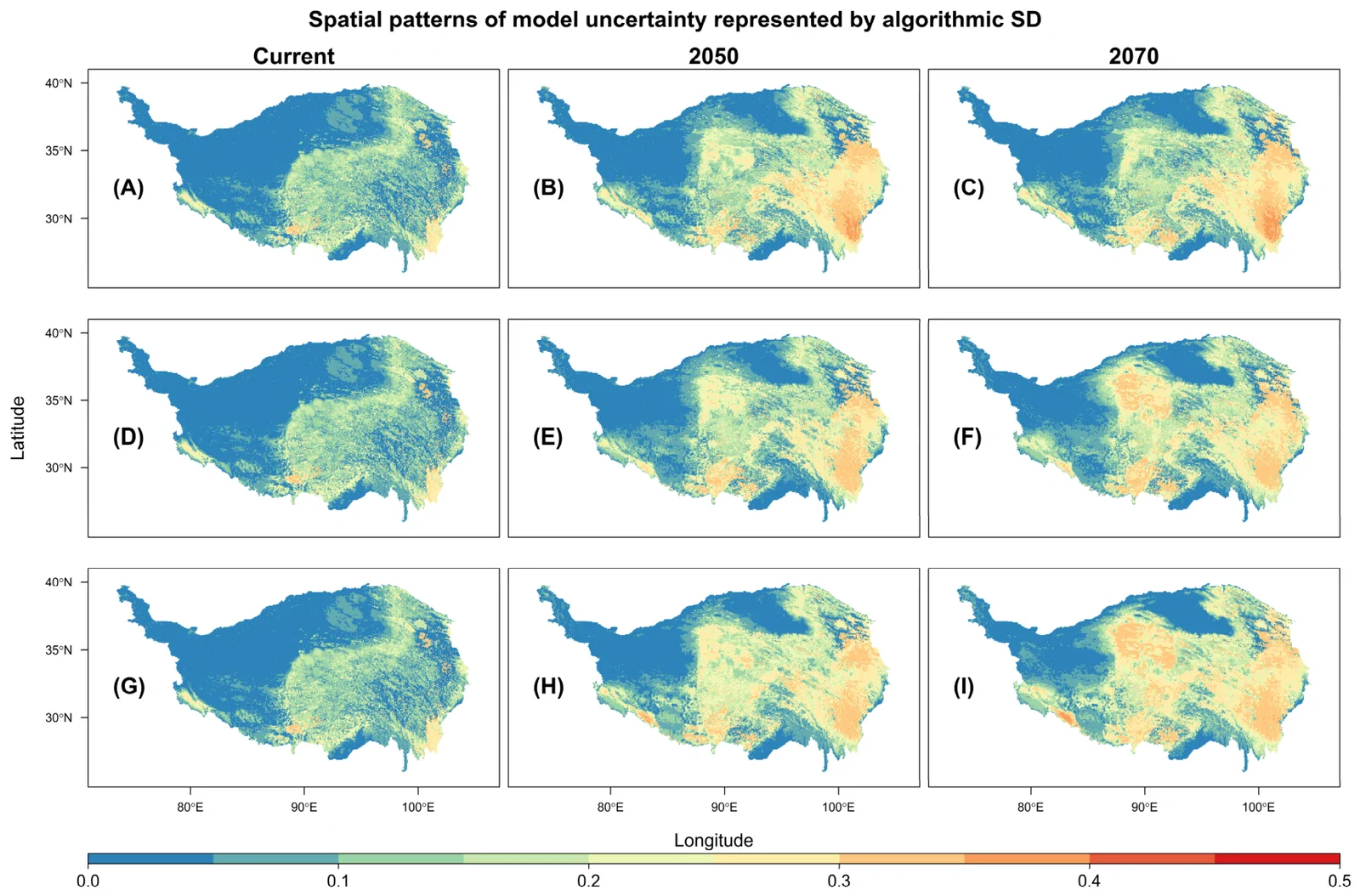
Spatial patterns of model uncertainty for the ensemble species distribution model of *Gymnaconitum gymnandrum* under current and future climate scenarios on the Qinghai–Tibet Plateau. (**A**,**D**,**G**) Current; (**B**) SSP1–2.6, 2050s; (**C**) SSP1–2.6, 2070s; (**E**) SSP3–7.0, 2050s; (**F**) SSP3–7.0, 2070s; (**H**) SSP5–8.5, 2050s; and (**I**) SSP5–8.5, 2070s.

**Figure 4:**
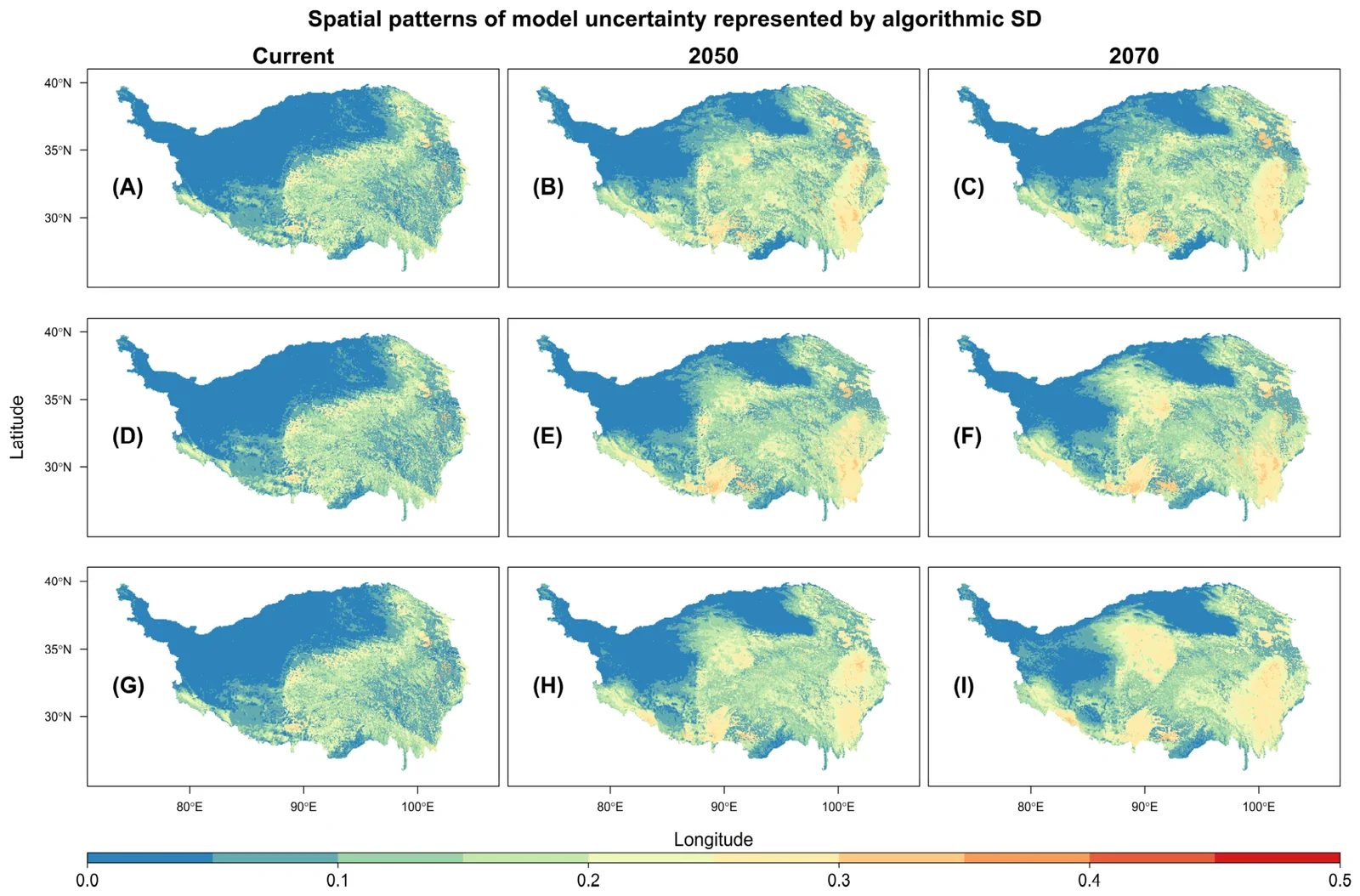
Spatial patterns of model uncertainty for the ensemble species distribution model of *Oxytropis kansuensis* under current and future climate scenarios on the Qinghai–Tibet Plateau. (**A**,**D**,**G**) Current; (**B**) SSP1–2.6, 2050s; (**C**) SSP1–2.6, 2070s; (**E**) SSP3–7.0, 2050s; (**F**) SSP3–7.0, 2070s; (**H**) SSP5–8.5, 2050s; and (**I**) SSP5–8.5, 2070s.

**Figure 5:**
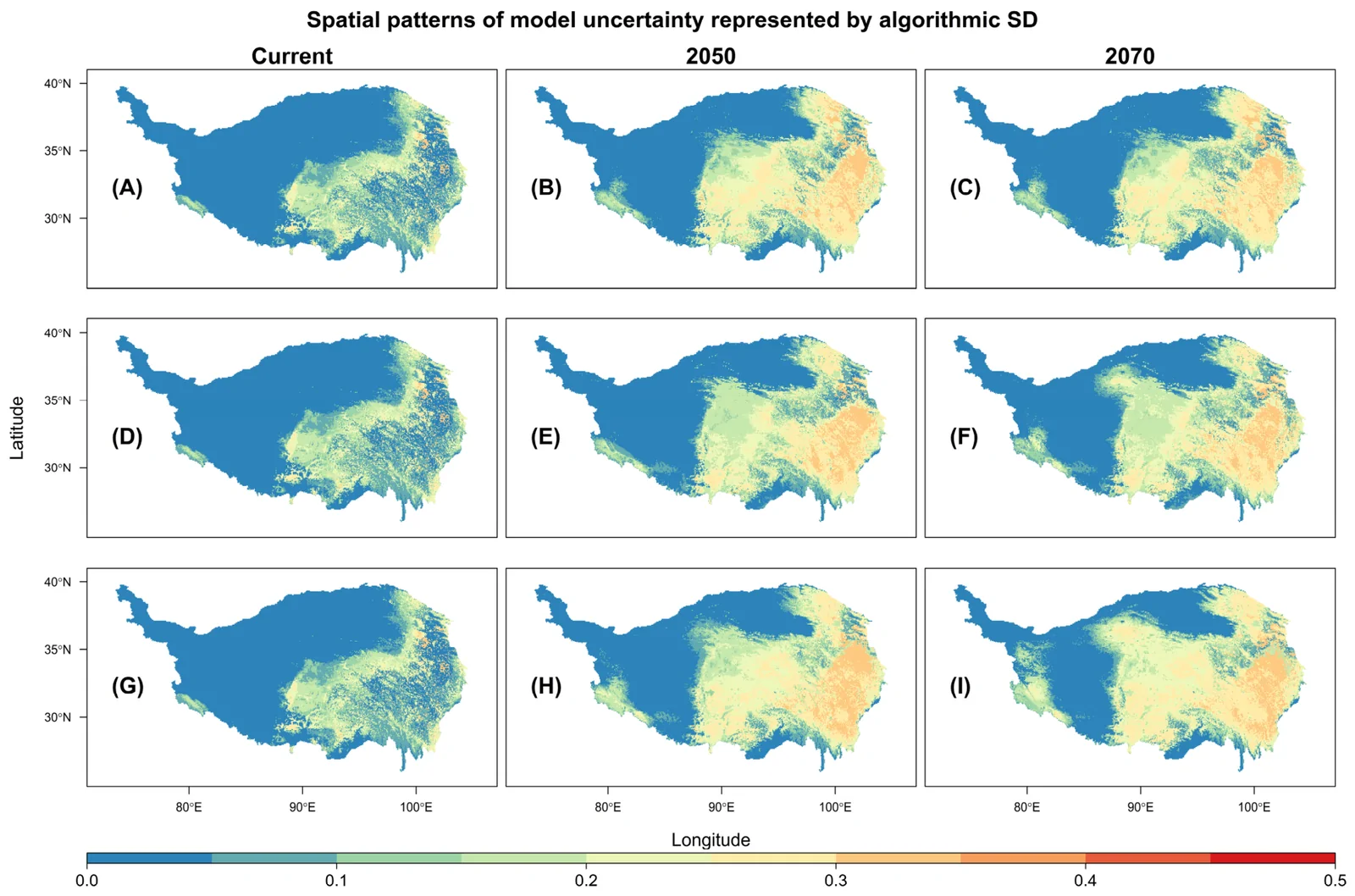
Spatial patterns of model uncertainty for the ensemble species distribution model of *Ligularia virgaurea* under current and future climate scenarios on the Qinghai–Tibet Plateau. (**A**,**D**,**G**) Current; (**B**) SSP1–2.6, 2050s; (**C**) SSP1–2.6, 2070s; (**E**) SSP3–7.0, 2050s; (**F**) SSP3–7.0, 2070s; (**H**) SSP5–8.5, 2050s; and (**I**) SSP5–8.5, 2070s.

Across future climate scenarios, the largest mean SD values for *Stellera chamaejasme* and *Thermopsis lanceolata* occurred under SSP3–7.0 during the 2070s, reaching 0.174 and 0.188, respectively. In contrast, *Gymnaconitum gymnandrum*, *Oxytropis kansuensis*, and *Ligularia virgaurea* exhibited their largest mean SD values under SSP5–8.5 during the 2070s, reaching 0.167, 0.139, and 0.154, respectively (Figure 1, Figure 2, Figure 3, Figure 4 and Figure 5; Table 1).

Spatially, higher among-algorithm variability occurred mainly along the margins of projected suitable habitats, whereas lower variability occurred within their interiors. These SD values describe disagreement among algorithm-specific predictions and do not quantify total predictive uncertainty or independently establish prediction accuracy.

### 2.2. Selection of Influencing Variables for Modeling

**Figure 6:**
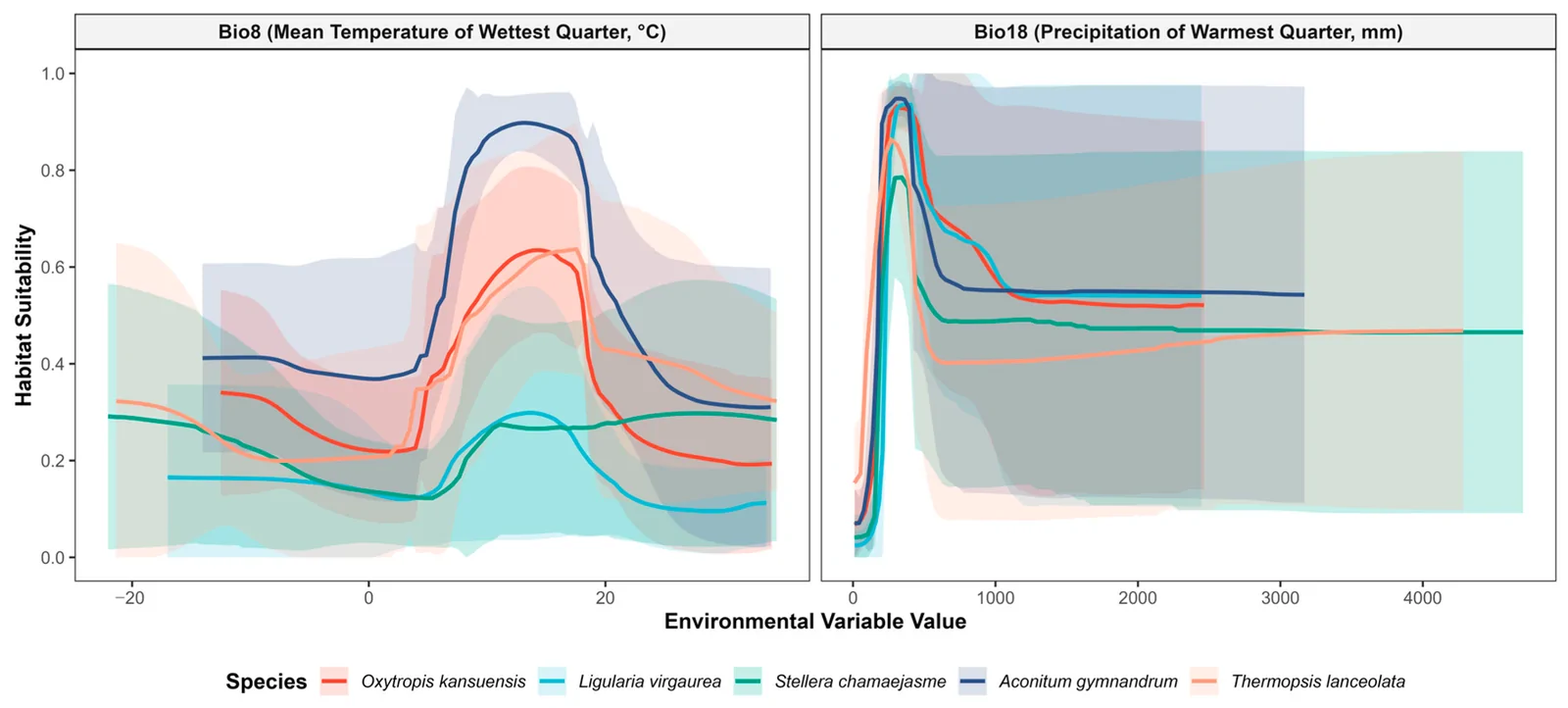
Response curves of habitat suitability to Bio8 and Bio18 for the five poisonous weed species. Note: Solid lines show the mean response across the seven algorithms. Shaded bands represent ±1 SD among algorithm-specific mean response curves at each predictor value and therefore indicate among-algorithm variability rather than confidence intervals. Other predictors were fixed at their median values.

A total of nine environmental variables were retained for model construction, and all variables had variance inflation factor (VIF) values below 5, indicating low multicollinearity among the retained predictors. Permutation-based variable importance is summarized in Table 2, while response curves for Bio8 and Bio18 are shown in Figure 6. Among the retained predictors, precipitation of the warmest quarter (Bio18) showed the highest mean importance across the five species (mean = 0.493), followed by mean temperature of the wettest quarter (Bio8) (mean = 0.204) and precipitation of the coldest quarter (Bio19) (mean = 0.156). Aspect and slope showed comparatively low mean importance values of 0.024 and 0.035, respectively. However, several predictors exhibited SDs that were large relative to their mean importance values, indicating substantial variation among individual model fits. For example, Bio8 had an importance of 0.295 ± 0.164 for *Thermopsis lanceolata*. Therefore, the mean importance values represent overall tendencies across the modelling framework rather than uniform agreement among algorithms, pseudo-absence datasets, and cross-validation repetitions. Overall, the results indicate that climatic predictors, particularly Bio18 and Bio8, were prominent contributors to model predictions, while the magnitude of their estimated importance varied among model realizations.

### 2.3. Current Distribution Patterns of Suitable Habitats for Five Poisonous Weed Species

**Figure 7:**
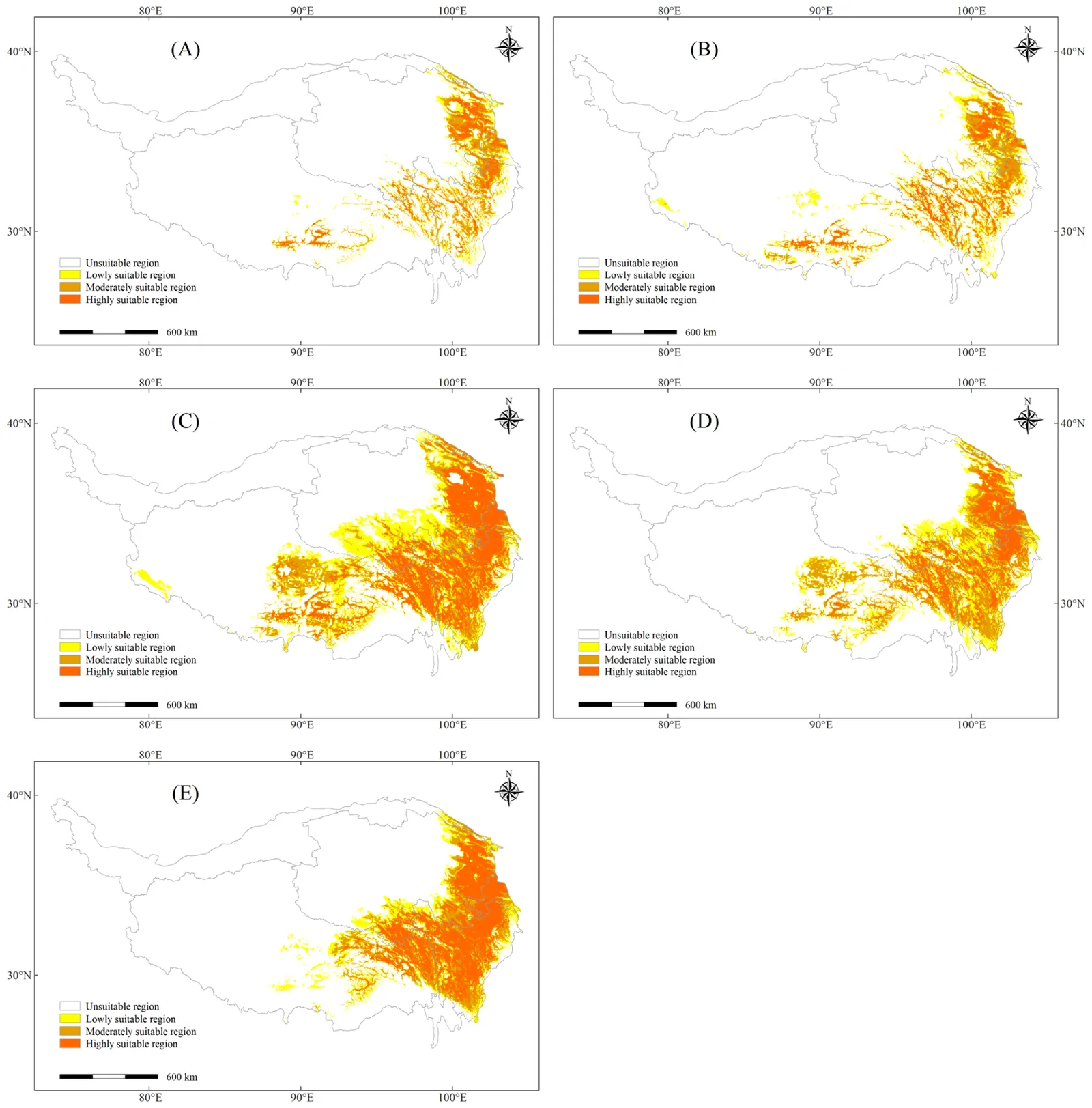
Spatial distribution of habitat suitability for five poisonous weed species on the Qinghai–Tibet Plateau under current climatic conditions. (**A**) *Stellera chamaejasme*; (**B**) *Thermopsis lanceolata*; (**C**) *Gymnaconitum gymnandrum*; (**D**) *Oxytropis kansuensis*; and (**E**) *Ligularia virgaurea*.

Under current climatic conditions, potentially suitable areas for the five species were concentrated mainly in the eastern and southeastern QTP, with additional patches along the southern margin; much of the western and northwestern plateau was classified as unsuitable (Figure 7). *Gymnaconitum gymnandrum* had the largest total suitable area (86.47 × 10^4^ km^2^), whereas *Stellera chamaejasme* had the smallest (28.13 × 10^4^ km^2^). The remaining species occupied intermediate positions under their respective species-specific classifications (Table 3).

Total suitable area and high-suitability area showed different interspecific rankings. Although *O. kansuensis* had a larger total suitable area than *L. virgaurea*, the latter had a larger high-suitability area. *G. gymnandrum* ranked highest for both measures. Spatially, suitable areas for *G. gymnandrum* and *O. kansuensis* extended farther into the south-central plateau, whereas those for *S. chamaejasme* and *T. lanceolata* were more restricted to the eastern and southern margins. These patterns distinguish the overall extent of projected suitability from the area assigned to the high-suitability class.

### 2.4. Future Changes in Potentially Suitable Habitats

**Figure 8:**
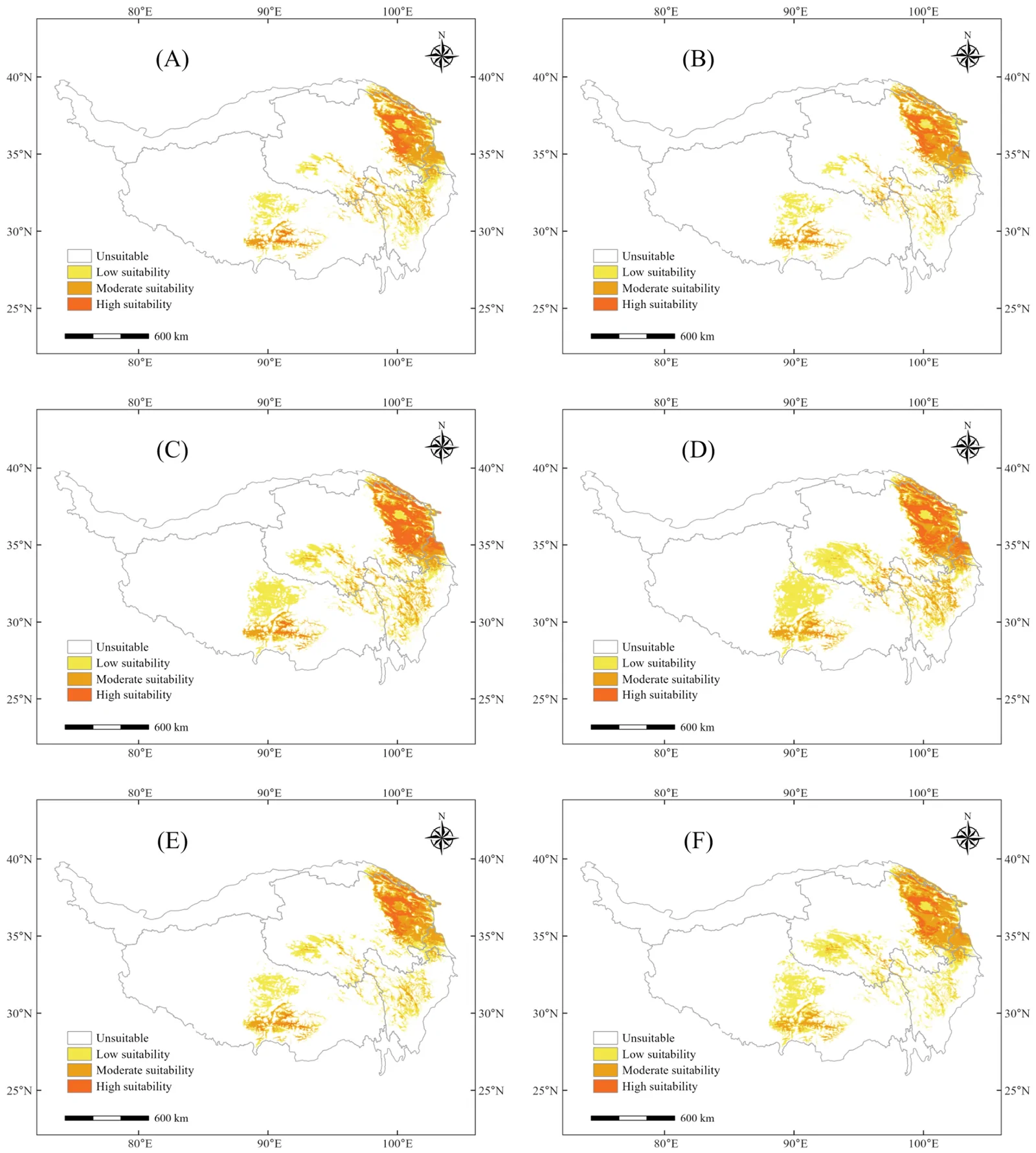
Predicted potential distribution of *Stellera chamaejasme* on the Qinghai–Tibet Plateau under different future climate change scenarios. (**A**) SSP1–2.6, 2050s; (**B**) SSP1–2.6, 2070s; (**C**) SSP3–7.0, 2050s; (**D**) SSP3–7.0, 2070s; (**E**) SSP5–8.5, 2050s; (**F**) SSP5–8.5, 2070s.

**Figure 9:**
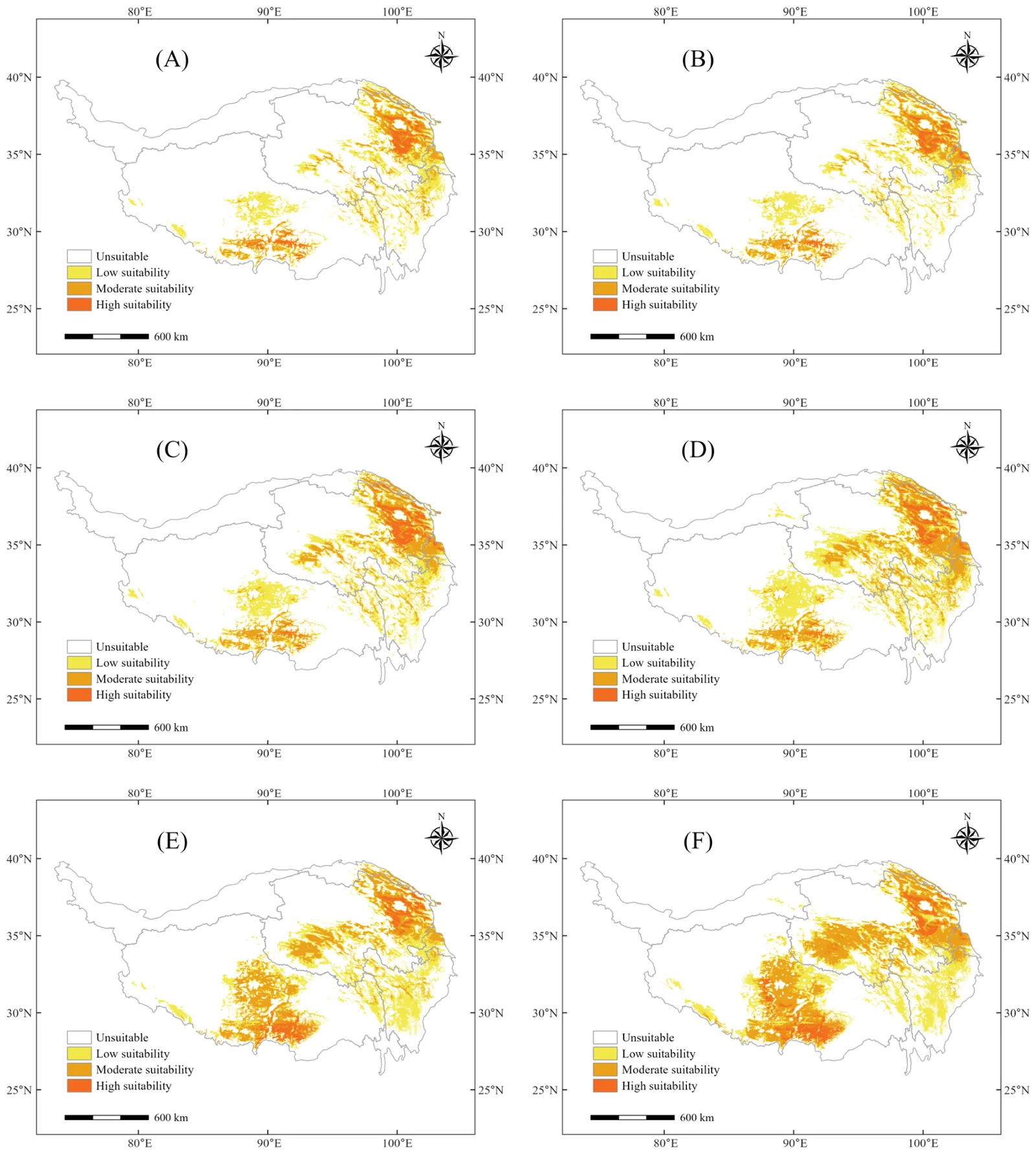
Predicted potential distribution of *Thermopsis lanceolata* on the Qinghai–Tibet Plateau under different future climate change scenarios. (**A**) SSP1–2.6, 2050s; (**B**) SSP1–2.6, 2070s; (**C**) SSP3–7.0, 2050s; (**D**) SSP3–7.0, 2070s; (**E**) SSP5–8.5, 2050s; (**F**) SSP5–8.5, 2070s.

**Figure 10:**
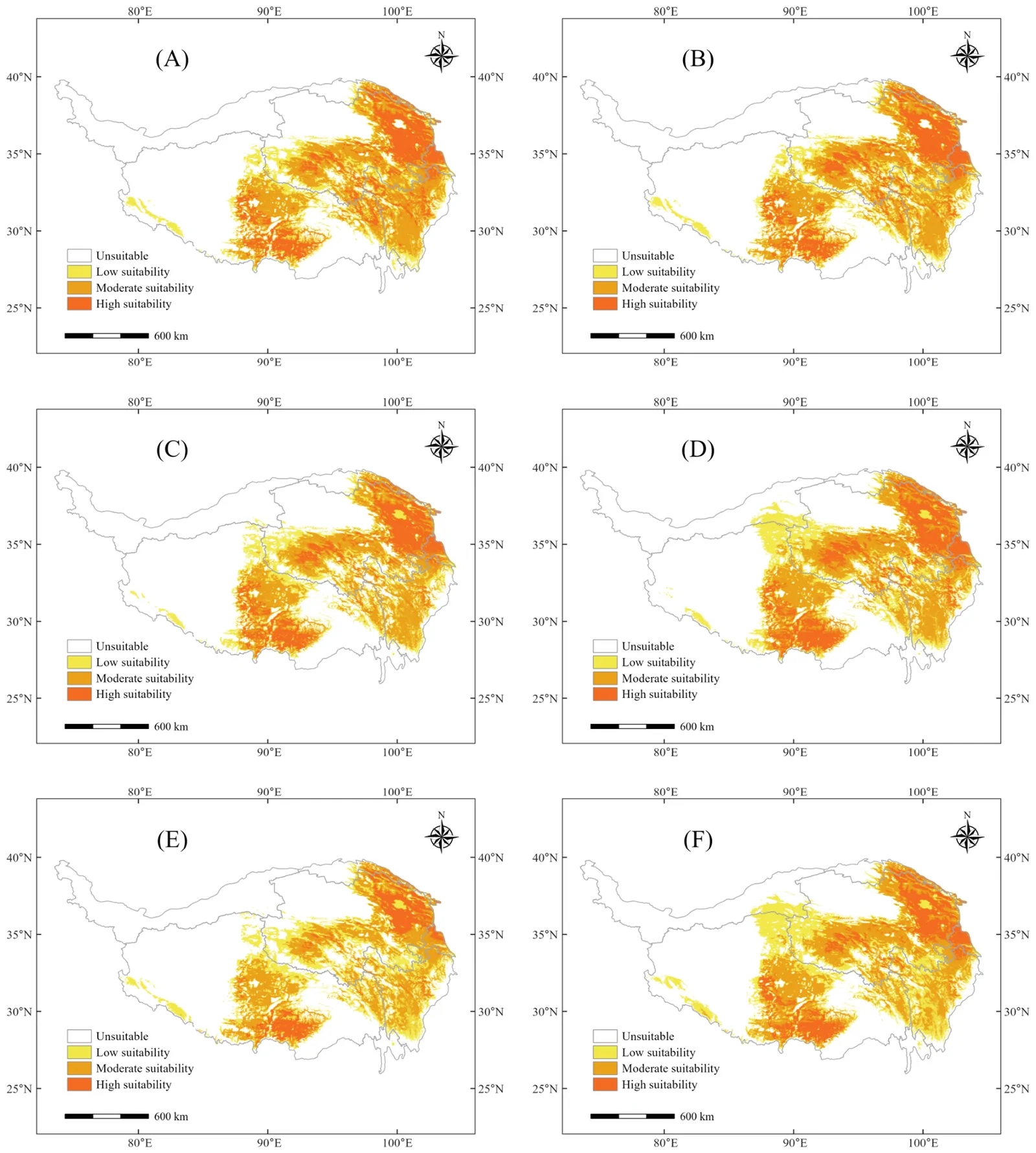
Predicted potential distribution of *Gymnaconitum gymnandrum* on the Qinghai–Tibet Plateau under different future climate change scenarios. (**A**) SSP1–2.6, 2050s; (**B**) SSP1–2.6, 2070s; (**C**) SSP3–7.0, 2050s; (**D**) SSP3–7.0, 2070s; (**E**) SSP5–8.5, 2050s; (**F**) SSP5–8.5, 2070s.

**Figure 11:**
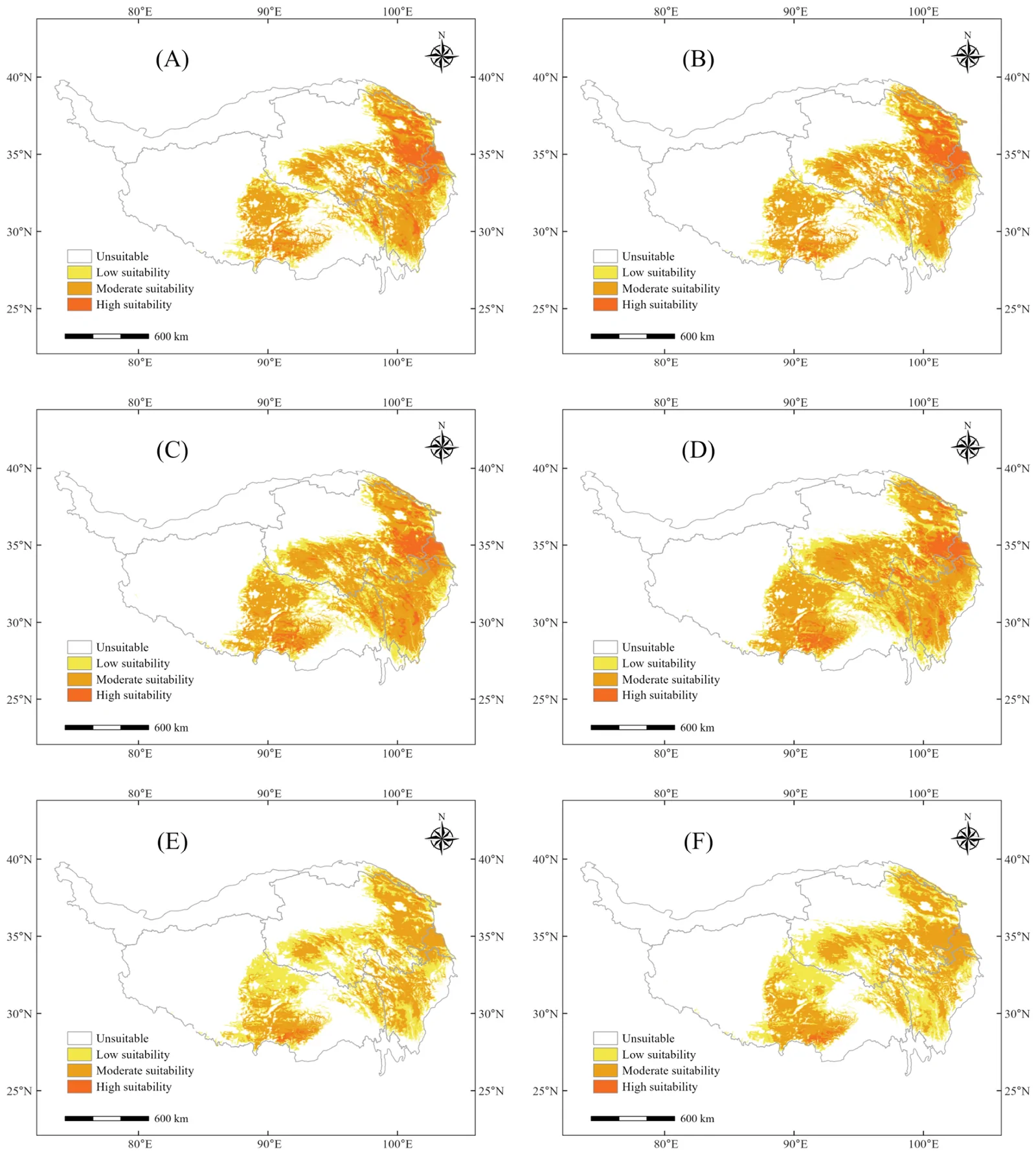
Predicted potential distribution of *Oxytropis kansuensis* on the Qinghai–Tibet Plateau under different future climate change scenarios. (**A**) SSP1–2.6, 2050s; (**B**) SSP1–2.6, 2070s; (**C**) SSP3–7.0, 2050s; (**D**) SSP3–7.0, 2070s; (**E**) SSP5–8.5, 2050s; (**F**) SSP5–8.5, 2070s.

**Figure 12:**
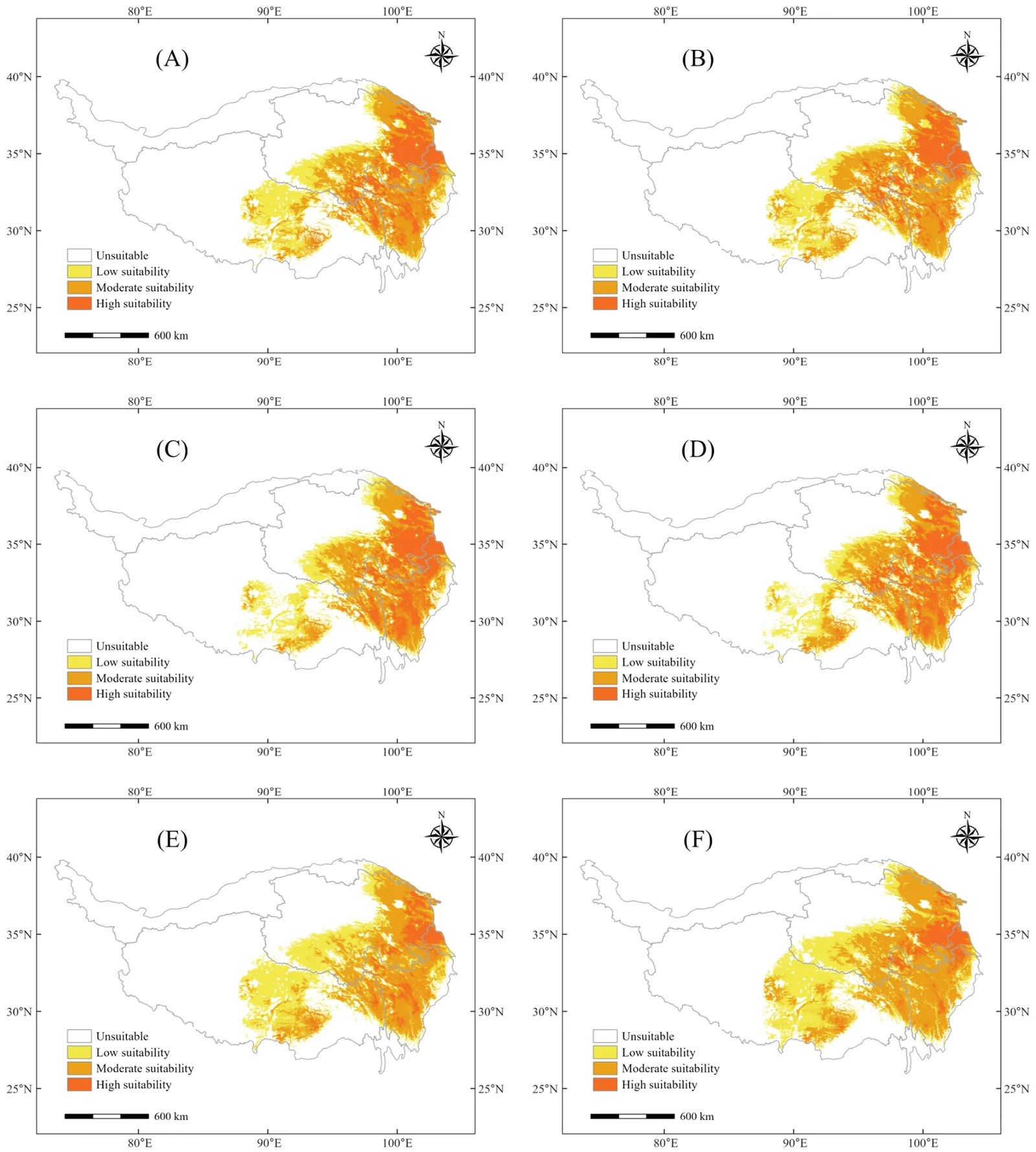
Predicted potential distribution of *Ligularia virgaurea* on the Qinghai–Tibet Plateau under different future climate change scenarios. (**A**) SSP1–2.6, 2050s; (**B**) SSP1–2.6, 2070s; (**C**) SSP3–7.0, 2050s; (**D**) SSP3–7.0, 2070s; (**E**) SSP5–8.5, 2050s; (**F**) SSP5–8.5, 2070s.

Under the BCC-CSM2-MR projections evaluated here, total potentially suitable area increased relative to the current baseline for all five species in every future scenario and period (Figure 8, Figure 9, Figure 10, Figure 11 and Figure 12; Table 4). *Gymnaconitum gymnandrum* retained the largest total suitable area throughout, whereas *Thermopsis lanceolata* showed the greatest relative increase, with its total suitable area more than doubling under SSP5–8.5 in the 2070s.

Expansion of total suitable area was accompanied by a shift towards low- and moderate-suitability classes. High-suitability area declined in every future projection for *G. gymnandrum*, *O. kansuensis*, *L. virgaurea*, and *T. lanceolata*. The clearest contrast occurred in *O. kansuensis*: despite an increase in total suitable area, its high-suitability area declined by approximately 94% under SSP5–8.5 in both future periods. *Stellera chamaejasme* differed from the other species, with high-suitability area increasing under SSP3–7.0 but decreasing under SSP1–2.6 and SSP5–8.5.

The temporal and scenario patterns were also species-dependent. Total suitable area increased from the 2050s to the 2070s for all five species under SSP3–7.0 and SSP5–8.5, whereas responses were mixed under SSP1–2.6. Higher forcing did not consistently correspond to greater suitable area: *S. chamaejasme* and *O. kansuensis* reached their largest projected areas under SSP3–7.0 rather than SSP5–8.5.

**Figure 13:**
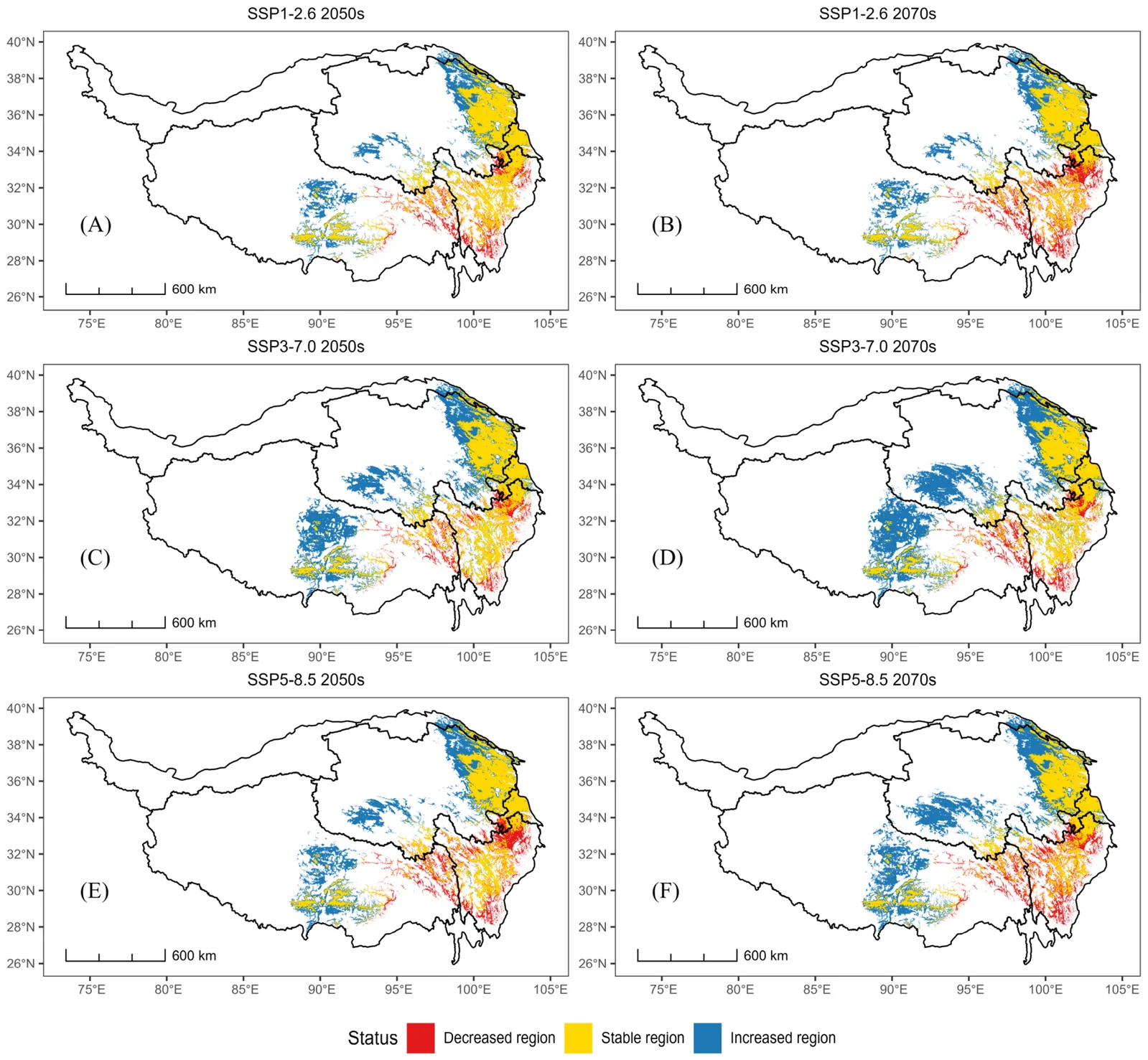
Changes in the suitable habitat of *Stellera chamaejasme* on the Qinghai–Tibet Plateau under future climate change scenarios. (**A**) SSP1–2.6, 2050s; (**B**) SSP1–2.6, 2070s; (**C**) SSP3–7.0, 2050s; (**D**) SSP3–7.0, 2070s; (**E**) SSP5–8.5, 2050s; (**F**) SSP5–8.5, 2070s.

**Figure 14:**
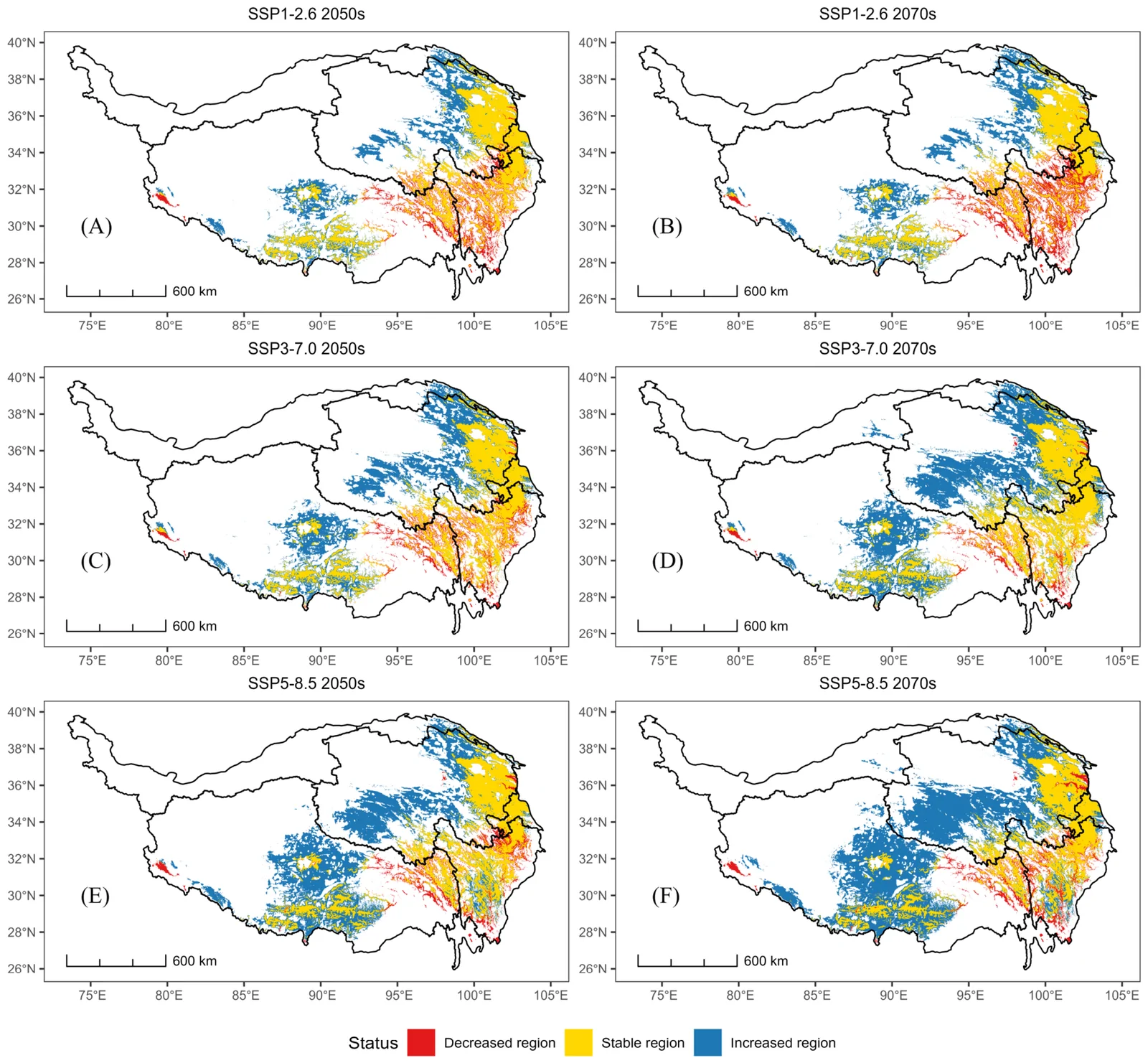
Changes in the suitable habitat of *Thermopsis lanceolata* on the Qinghai–Tibet Plateau under future climate change scenarios. (**A**) SSP1–2.6, 2050s; (**B**) SSP1–2.6, 2070s; (**C**) SSP3–7.0, 2050s; (**D**) SSP3–7.0, 2070s; (**E**) SSP5–8.5, 2050s; (**F**) SSP5–8.5, 2070s.

**Figure 15:**
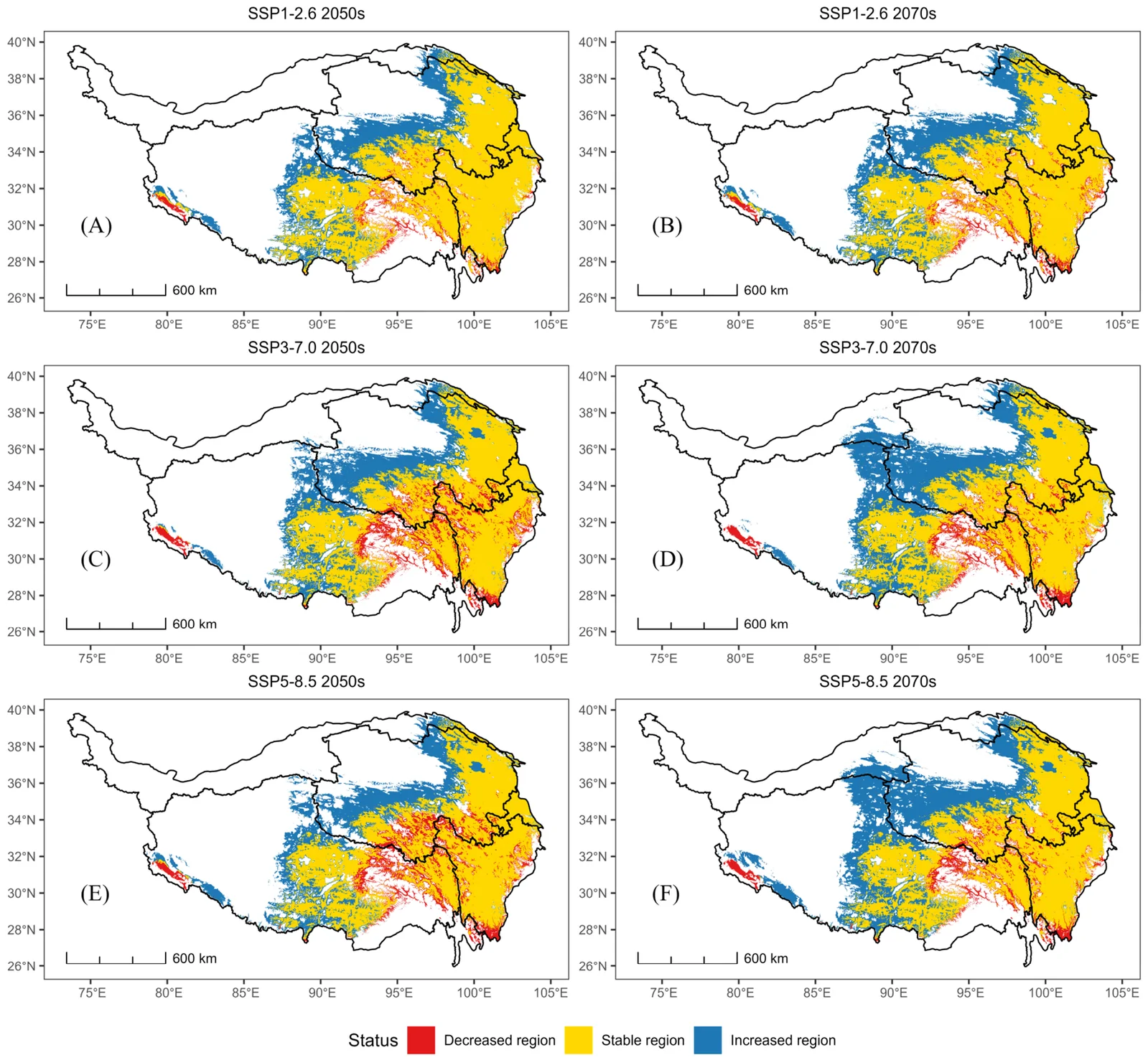
Changes in the suitable habitat of *Gymnaconitum gymnandrum* on the Qinghai–Tibet Plateau under future climate change scenarios. (**A**) SSP1–2.6, 2050s; (**B**) SSP1–2.6, 2070s; (**C**) SSP3–7.0, 2050s; (**D**) SSP3–7.0, 2070s; (**E**) SSP5–8.5, 2050s; (**F**) SSP5–8.5, 2070s.

**Figure 16:**
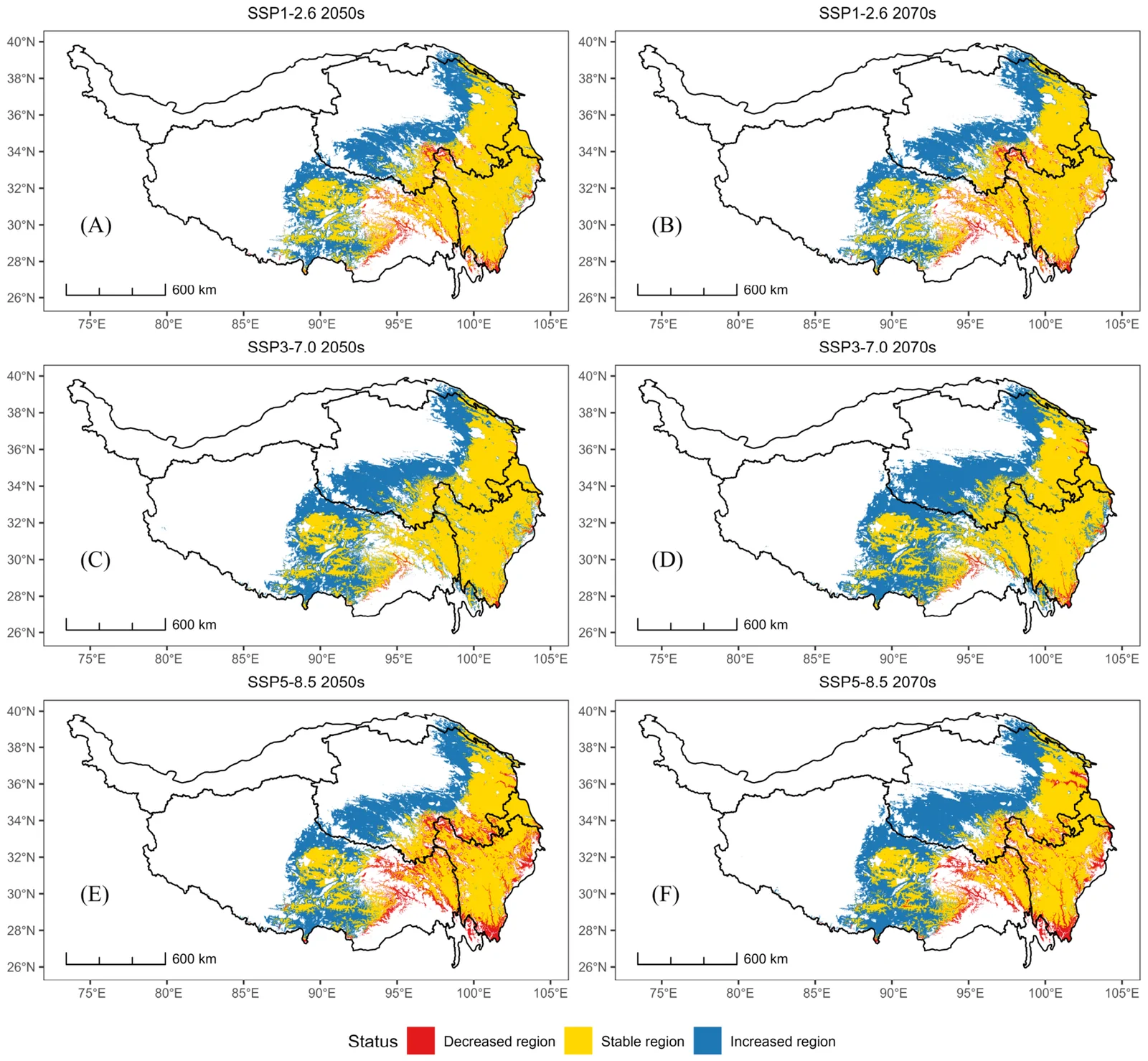
Changes in the suitable habitat of *Oxytropis kansuensis* on the Qinghai–Tibet Plateau under future climate change scenarios. (**A**) SSP1–2.6, 2050s; (**B**) SSP1–2.6, 2070s; (**C**) SSP3–7.0, 2050s; (**D**) SSP3–7.0, 2070s; (**E**) SSP5–8.5, 2050s; (**F**) SSP5–8.5, 2070s.

**Figure 17:**
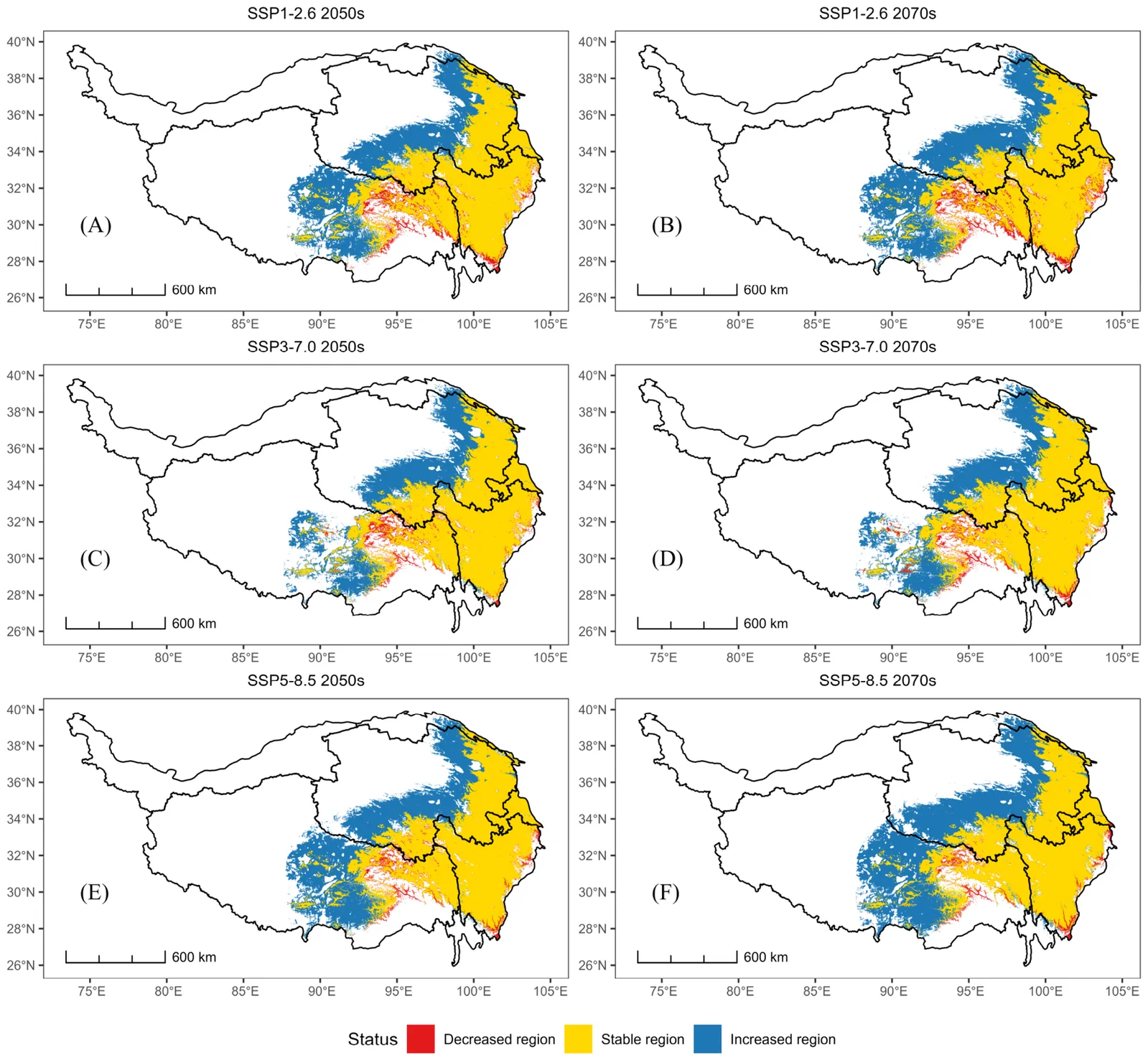
Changes in the suitable habitat of *Ligularia virgaurea* on the Qinghai–Tibet Plateau under future climate change scenarios. (**A**) SSP1–2.6, 2050s; (**B**) SSP1–2.6, 2070s; (**C**) SSP3–7.0, 2050s; (**D**) SSP3–7.0, 2070s; (**E**) SSP5–8.5, 2050s; (**F**) SSP5–8.5, 2070s.

The transition analysis showed that projected gains exceeded losses in every species–scenario–period combination, while substantial areas remained suitable relative to the current baseline (Figure 13, Figure 14, Figure 15, Figure 16 and Figure 17; Table 5). *G. gymnandrum* retained the largest stable suitable area throughout, and the largest individual gain was projected for *T. lanceolata* under SSP5–8.5 in the 2070s.

Together, these results show that net expansion of suitable area can coexist with losses of currently suitable locations and contraction of the high-suitability class. Total area, suitability-class composition, and spatial transitions therefore describe distinct aspects of the projected changes.

## 3. Discussion

### 3.1. Robustness and Reliability of Ensemble Projections

The EMwmean ensemble evaluation outputs yielded AUC values of 0.969–0.980 and TSS values of 0.818–0.884, indicating discrimination under the evaluation procedure used. This performance may partly reflect the pronounced environmental gradients across the QTP. TSS was used for model selection and ensemble weighting, while AUC provided an additional measure of discrimination [20]. These ensemble scores are not independent spatial hold-out estimates; the supplementary spatial validation assessed individual algorithms (Table S1). Neither high scores nor agreement among algorithms excludes overfitting or guarantees reliable future projections, which should therefore be interpreted cautiously [21].

### 3.2. Environmental Constraints on the Distribution of Poisonous Weeds

Our results showed that climatic variables were considerably more important than topographic variables in determining the potential distribution of poisonous weeds on the Qinghai–Tibetan Plateau (QTP). Among all predictors, precipitation of the warmest quarter (Bio18) contributed the most, followed by mean temperature of the wettest quarter (Bio8). This pattern indicates that hydrothermal conditions during the short alpine growing season are important environmental filters regulating species distributions, which is consistent with previous studies showing that climatic seasonality and hydrothermal conditions are major determinants of plant distribution patterns across the QTP [22,23]. The broad SD bands in parts of the Bio8 and Bio18 response curves indicate substantial among-algorithm variability; therefore, the mean curves should be interpreted as overall trends rather than uniform responses across algorithms.

The dominant role of Bio18 highlights the importance of summer water availability for alpine plants. Because most annual precipitation on the QTP occurs during the warmest months, Bio18 directly influences soil moisture, photosynthetic activity, nutrient uptake, biomass accumulation, and reproductive success. In alpine ecosystems with short growing seasons, even small changes in summer precipitation can substantially affect plant establishment and long-term population persistence, a pattern also observed in long-term precipitation manipulation experiments on alpine vegetation of the QTP [24]. Bio8 was the second most influential variable, reflecting the importance of suitable thermal conditions during the growing season. Warmer temperatures promote photosynthesis, extend the effective growing period, and facilitate flowering and seed maturation, whereas excessively low temperatures restrict plant growth and reproductive development. Growing-season temperature has likewise been identified as an important driver of plant productivity and species diversity across the QTP [25].

Species-specific responses suggest that different ecological strategies underlie these distribution patterns, supporting the environmental filtering hypothesis that species possessing different functional traits exhibit distinct responses to hydrothermal gradients in alpine grasslands [22]. *Oxytropis kansuensis* showed a high importance of Bio18, suggesting that although its deep taproot enhances drought tolerance in mature plants, summer precipitation may remain important for seedling establishment and population recruitment. *Ligularia virgaurea* exhibited the highest Bio18 importance among the five species, consistent with its preference for relatively moist alpine meadows, where continuous water availability may help sustain its large leaves and high transpiration demand [26]. *Stellera chamaejasme* also showed its highest variable importance for Bio18, while its extensive root system and clonal growth enable efficient resource acquisition and rapid colonization following grassland degradation, thereby increasing its competitive advantage under favorable moisture conditions [6]. *Thermopsis lanceolata* showed the highest Bio8 importance among the five species, although Bio18 remained its top-ranked predictor, suggesting that both growing-season temperature and summer precipitation may contribute importantly to its modeled habitat suitability. *Gymnaconitum gymnandrum* was also characterized by relatively high importance of Bio18 and Bio8, consistent with its adaptation to cool and moist alpine habitats where both water availability and suitable temperatures may influence flowering, seed development, and population persistence.

The relatively small contributions of slope and aspect indicate that topographic factors mainly influence local microhabitats, whereas regional climatic gradients determine broad-scale species distributions. As climate change continues to modify precipitation regimes and growing-season temperatures across the QTP, these hydrothermal changes are likely to alter competitive interactions within alpine grasslands, potentially facilitating the continued expansion of poisonous weeds into newly suitable habitats.

### 3.3. Current Potential Distribution Patterns and Highly Suitable Habitats of Five Poisonous Weed Species

Under current climatic conditions, the potentially suitable habitats of the five poisonous weed species were concentrated in the eastern and southeastern regions of the QTP, with additional suitable areas extending along its southern margin, corresponding mainly to alpine meadow ecosystems with relatively favorable hydrothermal conditions. In contrast, much of the western and northwestern plateau was classified as unsuitable. This spatial pattern suggests that the potential distributions of these species are associated with the pronounced temperature and moisture gradients across the QTP.

Previous modelling studies provide useful comparisons, although differences in predictors and classification methods limit direct comparison of area estimates. Liu et al. (2025), whose MaxEnt analysis included *S. chamaejasme*, identified the central and eastern alpine grasslands as important areas for the poisonous weeds examined and highlighted warmest-quarter precipitation alongside soil nitrogen [13]. Our results similarly emphasize summer precipitation, but do not account for soil effects. For *L. virgaurea*, Dong et al. (2023) identified suitable areas across the eastern QTP and surrounding regions, broadly agreeing with the geographic concentration found here [27]. However, their model also identified human footprint and soil nitrogen as important predictors, showing that climate-based suitability alone provides an incomplete account of this species’ distribution.

Field evidence offers a complementary comparison for *T. lanceolata*, *O. kansuensis*, and *G. gymnandrum*. A community study in the Qinghai Lake Basin recorded different patterns of occurrence and community niche breadth for these taxa along a grassland desertification gradient, with *G. gymnandrum* reported under its synonym *Aconitum gymnandrum* [28]. Such local differences provide ecological context for species-specific responses, but community niche breadth is not equivalent to regional climatic niche breadth. These observations therefore should not be treated as validation of the projected future changes.

The concentration of highly suitable habitats in the eastern and southeastern QTP may be associated with relatively suitable temperature and precipitation conditions, whereas low temperatures and limited moisture may restrict species establishment in the western and northwestern plateau. Similar environmental filtering effects have been reported for alpine grassland vegetation on the QTP [22,24,25]. The identified highly suitable areas may therefore represent priority regions for monitoring and managing poisonous weeds, particularly because the expansion of these species may contribute to changes in alpine grassland composition and ecological functioning [10,15].

### 3.4. Projected Changes in Habitat Suitability and Potential Distributions Under Future Climate Scenarios

Under the BCC-CSM2-MR projections evaluated here, total suitable habitat increased for all five poisonous weed species under all three SSPs, with habitat gains exceeding losses. Suitable habitats were projected to expand mainly into the northern and interior regions of the QTP, while localized contractions occurred in parts of the southern and southeastern plateau. This pattern may reflect the relaxation of low-temperature constraints in currently cold regions. Similar poleward and upslope distributional shifts have been reported for alpine plant species under climate warming [29,30].

The greatest relative increase in suitable area for *T. lanceolata* reflects both substantial projected gains and its comparatively small current suitable area. Relative change is calculated against the current baseline and therefore should not be interpreted as evidence that this species will attain the largest future distribution. Indeed, *G. gymnandrum* retained the largest absolute suitable area throughout the projections. For *T. lanceolata*, Bio8 had greater estimated importance than for the other four species, while Bio18 remained its highest-ranked predictor. This suggests that changes in growing-season temperature and precipitation warrant attention when interpreting its projections, but variable importance alone does not identify the direction or physiological mechanism of its response. The results therefore do not establish that warming directly enhances its growth, nitrogen fixation, or dispersal.

The coexistence of total suitable-area expansion and high-suitability contraction reflects changes in the spatial distribution of model scores rather than a contradiction. More locations can exceed the fixed binary suitability threshold while fewer exceed the higher boundary defining the high-suitability class. Thus, projected gains in low- and moderate-suitability areas can outweigh contraction of high-suitability areas. This distinction was particularly pronounced for *O. kansuensis* under SSP5–8.5. Because the class boundaries were held constant within each species, the pattern was not produced by recalculating thresholds separately for future maps. Nevertheless, its magnitude remains dependent on the chosen classification boundaries and should not be interpreted directly as a change in population abundance or persistence.

Given the importance of Bio18 and Bio8, changes in temperature and precipitation may increase modelled suitability in some locations while reducing it in others. However, this interpretation does not establish the physiological mechanisms underlying the projections. The high-suitability category is a descriptive model-score class, and its contraction should not be equated directly with declining abundance or population loss. Species responses were also not uniformly related to emission intensity, as the largest suitable areas were not always projected under the highest-emission scenario. This is consistent with previous evidence that climate change can alter alpine vegetation in complex and species-dependent ways [31,32].

Substantial areas of currently suitable habitat remained stable across the evaluated scenarios, while habitat gains varied among species, SSPs, and periods. Because future projections were based on a single GCM, they do not capture inter-model climate uncertainty and should be interpreted as conditional on BCC-CSM2-MR. Future studies should incorporate multiple GCMs to evaluate the robustness of projected habitat changes. In addition, dispersal limitations, biotic interactions, land-use change, and grazing disturbance were not included in the models [21,33]. Future monitoring should therefore consider both newly suitable habitats and areas projected to retain or lose high climatic suitability in vulnerable alpine grasslands [26,34].

### 3.5. Management Implications for the Control of Typical Poisonous Weeds on the Qinghai–Tibet Plateau

The projections can help prioritize monitoring of poisonous weeds by distinguishing newly suitable areas, persistent suitable areas, and areas of declining suitability. Their practical use requires combining the projected patterns with information on existing populations, grazed pastures, and livestock use. In particular, newly suitable areas in the northern and interior QTP may warrant early-detection surveys where they are accessible from existing populations and overlap with grazing activity. Operationally, the suitability and transition maps could be overlaid with pasture boundaries and known populations to select repeat-survey sites, where weed cover, recruitment, and grazing use would be recorded before management actions are chosen.

Monitoring priorities should reflect the different patterns projected for each species. The greatest relative expansion of *Thermopsis lanceolata* supports attention to potential establishment in newly suitable areas, whereas the large absolute suitable area of *Gymnaconitum gymnandrum* suggests a need for geographically extensive surveillance. For *Oxytropis kansuensis*, monitoring should cover both newly suitable areas and locations projected to lose high suitability. A decline in modelled suitability does not necessarily imply the disappearance of established populations or reduced livestock exposure. Field surveys should therefore record occurrence and abundance in both types of location.

Within the projected expansion fronts, including northern Tibet and the Qilian Mountains, combining remote sensing with targeted field surveys could help identify vegetation changes and confirm newly established populations [34]. In areas where persistent suitability coincides with observed weed abundance and pasture degradation, management options may include restoration of native forage vegetation, rotational grazing, and reduction in excessive grazing pressure [26,35]. These measures should be selected according to local vegetation and grazing conditions, as their effectiveness was not evaluated by the present models.

Finally, grazing decisions should incorporate locally observed weed phenology and species-specific toxicity information to reduce livestock exposure. Technical training and extension services for herders could support species identification, reporting of emerging populations, and appropriate local responses [35]. The suitability maps are therefore intended to guide monitoring and field assessment rather than prescribe control measures directly. Management interventions should be based on confirmed occurrence, abundance, and livestock exposure, supported by continued monitoring.

### 3.6. Study Limitations

The models characterize potential climatic suitability using climatic and topographic predictors, rather than realized species distributions. Soil properties, land use, grazing intensity, vegetation degradation, bare-ground availability, competition with palatable forage species, and local pasture management were not explicitly included. These factors may affect establishment, persistence, and local abundance, so areas projected to become climatically suitable may not necessarily be occupied or develop high weed abundance. Although slope and aspect were included, the approximately 1-km environmental resolution cannot fully represent fine-scale microtopography and pasture conditions. In addition, spatial filtering reduced local clustering of occurrence records, but residual geographic sampling bias was not formally quantified.

The projections assume that the ecological niches of the five poisonous weed species remain relatively stable over time, an assumption widely adopted in SDMs but increasingly discussed in recent studies on ecological niche conservatism [33]. However, species may exhibit adaptive responses, phenotypic plasticity, or evolutionary changes under long-term climate change, potentially altering their environmental tolerances and future distributions. These processes were not explicitly considered in the present study.

Although the Biomod2 ensemble framework effectively reduced uncertainty associated with individual algorithms and an algorithm uncertainty analysis was conducted, uncertainties related to future climate projections remain unavoidable. Different global climate models (GCMs), greenhouse gas emission pathways, and assumptions regarding future environmental conditions may lead to variation in predicted habitat suitability. Recent studies further indicate that uncertainty arising from consensus methods and climate models can substantially influence future SDM projections [36].

Furthermore, the projections presented in this study implicitly assume unlimited dispersal, meaning that all species are assumed to have access to newly climatically suitable habitats under future climate scenarios. In reality, dispersal limitations, geographical barriers, seed availability, biotic interactions, land-use change, grazing management, and local population processes may prevent species from occupying all climatically suitable areas. Therefore, the projected distributions should be interpreted as potential climatic suitability rather than actual future species distributions.

## 4. Materials and Methods

### 4.1. Study Area

The Qinghai–Tibet Plateau (26°00′–39°47′ N, 73°19′–104°47′ E) is located in southwestern China and encompasses Tibet, Qinghai, and parts of Xinjiang, Gansu, Sichuan, and Yunnan, covering approximately 2.5 million km^2^. With a mean elevation exceeding 4000 m, the plateau is characterized by complex topography and pronounced environmental heterogeneity. Its unique high-altitude alpine climate plays a fundamental role in shaping regional species distributions. Climatic conditions exhibit clear spatial gradients, with mean annual temperature decreasing from southeast to northwest and remaining below 0 °C across most areas, while annual precipitation declines from more than 2000 mm in the southeast to less than 50 mm in the northwest. Alpine meadows and alpine steppes dominate the region and are essential for biodiversity conservation and water-resource regulation.

### 4.2. Species Occurrence Data

Occurrence records were obtained from the Global Biodiversity Information Facility (GBIF) and the China Virtual Herbarium, with collection or observation dates ranging from 1970 to 2025. The GBIF occurrence downloads used in this study are documented by their DOI-based references [37,38,39,40,41]. Before filtering, the datasets contained 813, 1279, 877, 358, and 437 records for *Stellera chamaejasme*, *Thermopsis lanceolata*, *Gymnaconitum gymnandrum*, *Oxytropis kansuensis*, and *Ligularia virgaurea*, respectively. Taxonomic nomenclature was verified against Plants of the World Online (POWO), and synonymous occurrence records were harmonized to the currently accepted names. Duplicate records were removed, and one occurrence per 1-km grid cell was retained to reduce spatial clustering. After filtering, 231, 256, 190, 140, and 147 occurrence records were retained for *S. chamaejasme*, *T. lanceolata*, *G. gymnandrum*, *O. kansuensis*, and *L. virgaurea*, respectively. All occurrence records were standardized into geographic coordinates (latitude and longitude) and saved in CSV format.

**Figure 18:**
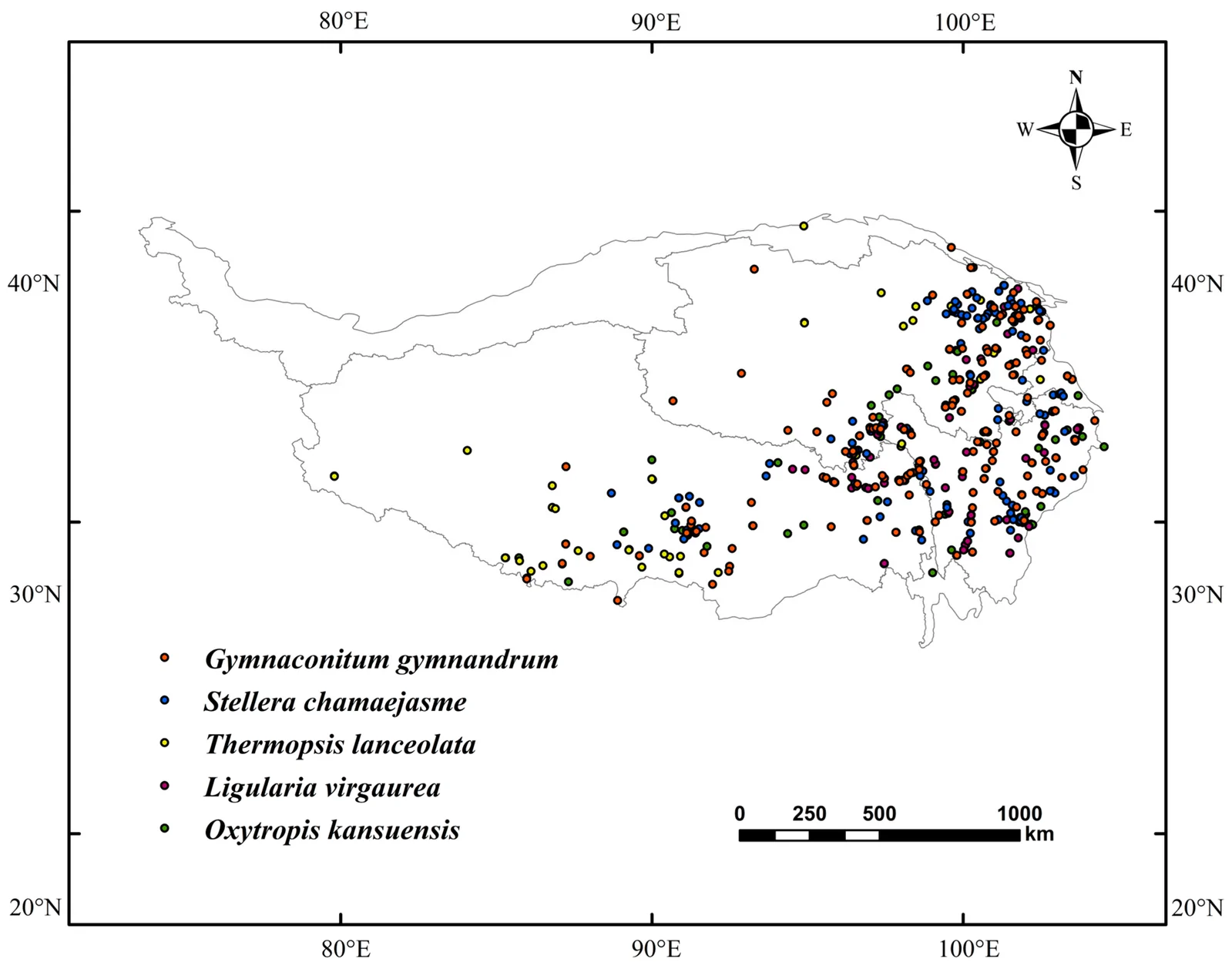
Spatial distribution of occurrence records of five poisonous weed species on the Qinghai–Tibet Plateau.

To reduce sampling bias and spatial autocorrelation caused by uneven sampling intensity, spatial thinning was conducted using ENMTools [42]. Considering that all environmental variables were standardized to a spatial resolution of approximately 1 km (30 arc-seconds), a 1 km × 1 km grid was applied for spatial filtering. When multiple occurrence records occurred within the same grid cell, only one record was randomly retained, whereas other clustered records were removed. This procedure reduced spatial aggregation of occurrence data and improved the reliability of species distribution modelling (Figure 18).

After spatial thinning and quality control, 231, 256, 190, 140, and 147 occurrence records were retained for *S. chamaejasme*, *T. lanceolata*, *G. gymnandrum*, *O. kansuensis*, and *L. virgaurea*, respectively. The filtered occurrence datasets were subsequently used as input data for the Biomod2 modelling framework [43].

### 4.3. Environmental Variables

Nineteen bioclimatic variables (Bio1–Bio19) representing current climatic conditions (1970–2000), together with elevation data, were obtained from the WorldClim database at a spatial resolution of 30 arc-seconds (approximately 1 km) [44]. Future bioclimatic variables for the 2050s (2041–2060) and 2070s (2061–2080) were obtained from WorldClim based on projections generated by the BCC-CSM2-MR global climate model under the Coupled Model Intercomparison Project Phase 6 (CMIP6) framework [45,46]. BCC-CSM2-MR was selected for its improved representation of East Asian climate and compatibility with the WorldClim scenarios used here. However, a single GCM cannot capture inter-model climate uncertainty. Three Shared Socioeconomic Pathway (SSP) scenarios were selected to represent different greenhouse gas emission trajectories: SSP1–2.6 (low-emission scenario), SSP3–7.0 (medium-to-high-emission scenario), and SSP5–8.5 (high-emission scenario). In addition to the bioclimatic variables, topographic variables, including elevation, slope, aspect, and the Terrain Ruggedness Index (TRI), were derived from the digital elevation model (DEM) using ArcGIS 10.8. All environmental variables were resampled to a common spatial resolution and aligned to the same raster grid to ensure spatial consistency in subsequent analyses. To minimize multicollinearity among environmental variables and improve model stability, variance inflation factor (VIF) analysis was conducted using the usdm package in R(version 4.5.2). Variables with VIF values ≥ 5 were sequentially removed using a stepwise procedure until all retained variables had VIF values < 5 [47,48,49,50]. Detailed descriptions of all environmental variables required for this study are provided in Table 6.

### 4.4. Uncertainty Analysis

To evaluate the variability introduced by different modeling algorithms, a spatially explicit uncertainty analysis was conducted for the ensemble model. Following recent studies on ensemble species distribution modelling and uncertainty quantification [51,52], we mapped prediction uncertainty by calculating the pixel-level standard deviation (SD) of the habitat suitability probabilities generated by the constituent single-algorithm models before ensemble averaging. Higher SD values represent greater variability and lower consensus among different algorithms, whereas lower SD values indicate higher model agreement [52,53]. This calculation was performed across all projection scenarios (Current, SSP1–2.6, SSP3–7.0, and SSP5–8.5) and time periods (2050s and 2070s) to evaluate how model uncertainty changes under future climate conditions, an approach increasingly adopted in recent ensemble SDM studies to characterize spatial uncertainty associated with climate change projections [54].

### 4.5. Model Development and Evaluation

Species distribution models (SDMs) were developed using Biomod2 version 3.5.1 in R to predict current and future potential distributions of the five poisonous weed species on the Qinghai–Tibet Plateau (QTP) [43,55]. For each species, five pseudo-absence datasets were generated (PA.nb.rep = 5), with the number of pseudo-absences approximating the occurrence count and rounded up to the nearest hundred [56,57]. The calibration background comprised the QTP and a 200-km outward buffer, providing a common regional extent that included surrounding environmental conditions. Comparable buffering has been used in Himalayan plant distribution modelling [58], with additional precedent from background-distance experiments [59]. Pseudo-absences were randomly sampled from cells with valid environmental data, excluding known occurrence locations. This background represented an operational sampling extent rather than a species-specific dispersal limit.

Seven algorithms were used: Generalized Linear Model (GLM), Generalized Boosting Model (GBM), Classification Tree Analysis (CTA), Artificial Neural Network (ANN), Surface Range Envelope (SRE), Flexible Discriminant Analysis (FDA), and Random Forest (RF). Data were randomly divided into training (75%) and evaluation (25%) subsets [60]. Five partitions for each of the five pseudo-absence datasets produced 25 replicates per algorithm, accounting for variation in pseudo-absence selection and data partitioning [57,60].

Model discrimination was evaluated using the True Skill Statistic (TSS) and the Area Under the Receiver Operating Characteristic Curve (AUC) [61,62]. These metrics do not directly assess probability calibration or independently establish spatial transferability or predictive robustness. Replicates with TSS ≥ 0.75 were retained for ensemble modelling, and predictions were combined using the TSS-weighted mean approach (EMwmean). In addition to the 75%/25% random-partition evaluation, five-fold spatial block cross-validation was conducted for each species using the blockCV package [63]. Hexagonal blocks were generated using the rows_cols setting of c(10, 10), without specifying a fixed metric block size. For each species, a single pseudo-absence dataset was generated, with the number of pseudo-absences determined by rounding the occurrence count upward to the next hundred. Spatial blocks were randomly assigned to five folds, and the allocation was repeated until both presence and pseudo-absence records were represented in every training and evaluation subset. This acceptance criterion was based solely on the representation of both response classes, rather than model performance. Fold assignments were supplied to Biomod2 through DataSplitTable. For each species–algorithm combination, held-out TSS and AUC were averaged across all five folds, without excluding low-performing folds. Across the 35 species–algorithm combinations, the mean ± SD of these five-fold mean scores was 0.703 ± 0.106 for TSS and 0.871 ± 0.063 for AUC. These SDs describe variation among species–algorithm mean scores, rather than variation among individual folds. The spatial validation results assess individual algorithms and are not interpreted as independent validation of ensembles formed by merging models across folds. Ensemble models generated continuous suitability maps for current and future climates [64].

Continuous predictions were converted into binary maps using species-specific thresholds that maximized TSS [61], equivalent to maximizing sensitivity plus specificity [65]. The EMwmean thresholds were 0.688 for *Stellera chamaejasme*, 0.555 for *Oxytropis kansuensis*, 0.502 for *Ligularia virgaurea*, 0.414 for *Gymnaconitum gymnandrum*, and 0.584 for *Thermopsis lanceolata*. Exported rasters on the 0–1000 scale were divided by 1000 before applying these thresholds; cells at or above the threshold were classified as suitable. Because threshold choice affects estimated range changes [66,67], each species’ threshold remained fixed across all scenarios and periods.

For descriptive mapping, the suitable interval [t, 1], where t denotes the binary threshold, was divided into three equal classes. For suitability score s, low suitability was defined as t ≤ s < t + (1 − t)/3, moderate suitability as t + (1 − t)/3 ≤ s < t + 2(1 − t)/3, and high suitability as t + 2(1 − t)/3 ≤ s ≤ 1. These boundaries remained fixed within each species and represented descriptive categories rather than independently validated biological thresholds.

Predictions were cropped and masked to the original QTP boundary, excluding the external buffer and missing cells. Class areas were calculated by summing cell-specific areas and reported in 10^4^ km^2^, with total suitable area comprising all three classes. Habitat gains, losses, and stable suitable areas were derived by comparing future binary maps with a common current baseline for each species.

## 5. Conclusions

Using the Biomod2 ensemble modelling framework, this study assessed the current and future potential climatic suitability for five poisonous weed species on the Qinghai–Tibet Plateau using climatic and topographic predictors, with future projections based on a single GCM, BCC-CSM2-MR, under three SSP scenarios. The study further evaluated the effects of key environmental variables on species distributions and quantified changes in habitat suitability across future periods. The main findings are as follows: (1) The ensemble models showed strong discriminatory performance, with TSS values ranging from 0.818 to 0.884. (2) Precipitation of the warmest quarter (Bio18) and mean temperature of the wettest quarter (Bio8) had the highest average importance across the five species, indicating that hydrothermal conditions are important in shaping their potential distributions on the QTP. (3) Under current climatic conditions, potentially suitable habitats were mainly concentrated in the eastern and southeastern QTP, with additional suitable areas along its southern margin, although their extent and spatial configuration differed among species. *Gymnaconitum gymnandrum* had the largest total and most highly suitable areas. (4) Under the BCC-CSM2-MR projections, total potentially suitable habitat increased for all five species across the three SSPs, with habitat gains exceeding losses. *Gymnaconitum gymnandrum* retained the largest total suitable area, whereas *Thermopsis lanceolata* exhibited the greatest relative increase. However, increases in total suitable area did not consistently correspond to increases in highly suitable habitat. In particular, the highly suitable habitat of *Oxytropis kansuensis* declined by approximately 94% under SSP5–8.5 in both future periods despite an increase in its total suitable area. Overall, the projections indicate changes in the extent and spatial distribution of modelled climatic suitability, including expansion of total suitable area alongside contraction of highly suitable area for some species. These findings highlight the need to consider both total suitable area and suitability-class composition in ecological risk assessment, targeted monitoring, and adaptive grassland management. These results are conditional on BCC-CSM2-MR and do not capture inter-GCM uncertainty. Because soil conditions, grazing, land use, biotic interactions, local management, and dispersal limitations were not explicitly modelled, changes in climatic suitability should not be interpreted as realized range shifts, changes in weed abundance, or changes in livestock exposure. Their management application therefore requires field verification.

## Figures and Tables

**Table 1:** Mean algorithm uncertainty (mean SD).

Species	2050s				2070s		
	Current	SSP1–2.6	SSP3–7.0	SSP5–8.5	SSP1–2.6	SSP3–7.0	SSP5–8.5
*Stellera chamaejasme*	0.100	0.143	0.162	0.152	0.144	0.174	0.167
*Thermopsis lanceolata*	0.142	0.169	0.180	0.162	0.171	0.188	0.174
*Gymnaconitum gymnandrum*	0.087	0.140	0.136	0.149	0.140	0.151	0.167
*Oxytropis kansuensis*	0.093	0.126	0.122	0.128	0.128	0.130	0.139
*Ligularia virgaurea*	0.073	0.128	0.125	0.141	0.128	0.132	0.154

**Table 2:** Permutation-based variable importance of environmental predictors for the five poisonous weed species.

Variables	*Oxytropis* *kansuensis*	*Ligularia* *virgaurea*	*Stellera* *chamaejasme*	*Gymnaconitum* *gymnandrum*	*Thermopsis* *lanceolata*
Bio2	0.064 ± 0.072	0.086 ± 0.092	0.034 ± 0.054	0.087 ± 0.079	0.072 ± 0.070
Bio3	0.073 ± 0.079	0.042 ± 0.053	0.045 ± 0.061	0.054 ± 0.069	0.120 ± 0.088
Bio8	0.204 ± 0.131	0.150 ± 0.120	0.171 ± 0.105	0.200 ± 0.141	0.295 ± 0.164
Bio14	0.071 ± 0.082	0.057 ± 0.071	0.041 ± 0.059	0.037 ± 0.051	0.043 ± 0.058
Bio15	0.085 ± 0.084	0.118 ± 0.103	0.061 ± 0.074	0.070 ± 0.086	0.082 ± 0.093
Bio18	0.534 ± 0.164	0.573 ± 0.143	0.496 ± 0.123	0.499 ± 0.141	0.360 ± 0.168
Bio19	0.124 ± 0.128	0.187 ± 0.153	0.231 ± 0.151	0.140 ± 0.149	0.099 ± 0.119
Aspect	0.031 ± 0.046	0.024 ± 0.040	0.021 ± 0.033	0.020 ± 0.033	0.026 ± 0.040
Slope	0.039 ± 0.064	0.036 ± 0.067	0.031 ± 0.054	0.031 ± 0.059	0.039 ± 0.063

**Note:** Values are presented as mean ± SD of permutation-based variable-importance estimates across 175 cross-validation model fits for each species and predictor (7 algorithms × 5 pseudo-absence datasets × 5 cross-validation repetitions). Models fitted to the full dataset were excluded. The SD represents the combined model-to-model variation among algorithms, pseudo-absence datasets, and cross-validation repetitions and should not be interpreted as variability attributable to any single component.

**Table 3:** Current habitat area of five poisonous weed species on the QTP.

Species	Low (×10^4^ km^2^)	Moderate (×10^4^ km^2^)	High (×10^4^ km^2^)	Total Suitable (×10^4^ km^2^)	High (%)	Total (%)
*Stellera chamaejasme*	9.34	12.35	6.44	28.13	2.48	10.85
*Thermopsis lanceolata*	14.08	15.00	8.00	37.09	3.09	14.31
*Gymnaconitum gymnandrum*	29.14	25.21	32.11	86.47	12.39	33.36
*Oxytropis kansuensis*	21.35	27.00	16.45	64.8	6.35	25.00
*Ligularia virgaurea*	14.36	17.90	28.67	60.94	11.06	23.51

**Table 4:** Current and projected areas of potentially suitable habitats for the five poisonous weed species on the Qinghai–Tibet Plateau under different climate scenarios.

Species	Suitability Level (×10^4^ km^2^)	2050				2070		
		Current	SSP1–2.6	SSP3–7.0	SSP5–8.5	SSP1–2.6	SSP3–7.0	SSP5–8.5
*Stellera chamaejasme*	Low suitability	9.34	16.63	22.34	19.44	15.32	27.07	24.98
	Moderate suitability	12.35	14.11	13.36	12.71	13.42	15.36	14.21
	High suitability	6.44	4.28	8.89	4.21	3.77	7.11	3.14
	Total suitable habitat	28.13	35.02	44.60	36.36	32.50	49.54	42.33
*Thermopsis lanceolata*	Low suitability	14.08	27.77	32.11	36.80	26.15	42.88	39.17
	Moderate suitability	15.00	14.12	19.81	27.49	14.38	26.06	37.75
	High suitability	8.00	3.88	4.87	5.59	3.44	4.22	7.29
	Total suitable habitat	37.09	45.77	56.79	69.89	43.96	73.16	84.21
*Gymnaconitum gymnandrum*	Low suitability	29.14	31.26	33.51	42.20	32.23	38.19	47.48
	Moderate suitability	25.21	52.50	49.43	46.11	52.80	53.98	53.90
	High suitability	32.11	27.00	22.65	14.39	26.51	26.75	22.00
	Total suitable habitat	86.47	110.77	105.59	102.70	111.54	118.91	123.39
*Oxytropis kansuensis*	Low suitability	21.35	26.58	31.62	45.96	27.82	35.79	48.96
	Moderate suitability	27.00	51.09	62.26	39.27	51.89	63.90	45.27
	High suitability	16.45	12.32	11.21	0.95	10.91	12.09	0.98
	Total suitable habitat	64.80	89.99	105.08	86.19	90.61	111.77	95.21
*Ligularia virgaurea*	Low suitability	14.36	30.24	25.90	40.18	29.97	26.30	41.78
	Moderate suitability	17.90	38.14	34.78	43.89	42.23	35.25	50.77
	High suitability	28.67	20.36	22.63	10.60	18.43	23.05	10.53
	Total suitable habitat	60.94	88.74	83.31	94.68	90.62	84.60	103.08

**Table 5:** Habitat transition statistics of potentially suitable habitats relative to the current climate.

Species	Dynamics (×10^4^ km^2^)	2050s			2070s		
		SSP1–2.6	SSP3–7.0	SSP5–8.5	SSP1–2.6	SSP3–7.0	SSP5–8.5
*Stellera chamaejasme*	Gain	12.29	21.21	16.23	12.39	26.73	22.16
	Loss	5.40	4.74	7.99	8.01	5.32	7.96
	Stable	22.73	23.39	20.14	20.12	22.81	20.17
*Thermopsis lanceolata*	Gain	17.58	26.70	39.41	18.71	40.33	54.46
	Loss	8.90	6.99	6.60	11.83	4.25	7.34
	Stable	28.18	30.09	30.48	25.25	32.83	29.74
*Gymnaconitum gymnandrum*	Gain	30.40	33.02	30.94	32.72	43.38	47.15
	Loss	6.09	13.89	14.70	7.65	10.94	10.22
	Stable	80.37	72.57	71.77	78.82	75.53	76.24
*Oxytropis kansuensis*	Gain	30.59	41.57	33.76	32.43	49.06	41.46
	Loss	5.40	1.28	12.38	6.61	2.09	11.04
	Stable	59.40	63.52	52.42	58.18	62.71	53.76
*Ligularia virgaurea*	Gain	32.88	26.40	37.42	35.72	27.84	45.09
	Loss	5.07	4.03	3.69	6.03	4.18	2.95
	Stable	55.87	56.91	57.25	54.90	56.76	57.99

**Table 6:** Information on environmental variables.

Variables	Description	Source	Units
Bio1	Annual Mean Temperature	WorldClim (https://www.worldclim.org/)	°C
Bio2	Mean Diurnal Range	WorldClim (https://www.worldclim.org/)	°C
Bio3	Isothermality	WorldClim (https://www.worldclim.org/)	%
Bio4	Temperature Seasonality	WorldClim (https://www.worldclim.org/)	-
Bio5	Max Temperature of Warmest Month	WorldClim (https://www.worldclim.org/)	°C
Bio6	Min Temperature of Coldest Month	WorldClim (https://www.worldclim.org/)	°C
Bio7	Temperature Annual Range	WorldClim (https://www.worldclim.org/)	°C
Bio8	Mean Temperature of Wettest Quarte	WorldClim (https://www.worldclim.org/)	°C
Bio9	Mean Temperature of Driest Quarter	WorldClim (https://www.worldclim.org/)	°C
Bio10	Mean Temperature of Warmest Quarter	WorldClim (https://www.worldclim.org/)	°C
Bio11	Mean Temperature of Coldest Quarter	WorldClim (https://www.worldclim.org/)	°C
Bio12	Annual Precipitation	WorldClim (https://www.worldclim.org/)	mm
Bio13	Precipitation of Wettest Month	WorldClim (https://www.worldclim.org/)	mm
Bio14	Precipitation of Driest Month	WorldClim (https://www.worldclim.org/)	mm
Bio15	Precipitation Seasonality	WorldClim (https://www.worldclim.org/)	-
Bio16	Precipitation of Wettest Quarter	WorldClim (https://www.worldclim.org/)	mm
Bio17	Precipitation of Driest Quarter	WorldClim (https://www.worldclim.org/)	mm
Bio18	Precipitation of Warmest Quarter	WorldClim (https://www.worldclim.org/)	mm
Bio19	Precipitation of coldest quarter	WorldClim (https://www.worldclim.org/)	mm
Alt.	Altitude	DEM	m
Asp.	Aspect	DEM	-
Slop.	Slope	DEM	-
TRI	Terrain Ruggedness Index	DEM	m

## Data Availability

The processed occurrence data and analysis scripts supporting this study are available from the corresponding author upon reasonable request. The original occurrence records were obtained from the Global Biodiversity Information Facility (GBIF) and the China Virtual Herbarium.

## Supplementary Materials

The following supporting information can be downloaded at: https://www.mdpi.com/article/10.3390/plants15203219/s1, Table S1 Model evaluation results; Table S2 Comparison of random and spatial validation.
