# Supplementary material for: Predicting the Potential Climatic Suitability of Typical Poisonous Weeds on the Qinghai–Tibet Plateau Under Climate Change Using Biomod2 and a Single Global Climate Model
Source: Plants (Basel). 2026 Oct 20;15(20):3219. doi: 10.3390/plants15203219 (PMC13708998; doi:10.3390/plants15203219)
Supplement: Supplementary file 1 [file plants-15-03219-s001.zip › plants-4509353-supplementary.pdf]

Supplementary Materials

Predicting the potential climatic suitability of typical poisonous weeds on the Qinghai–Tibet Plateau under climate change using biomod2 and a single global climate model

Ruiqi Yang[1,2], Hu Ma[2], Wenwen Guo[2], Siqing Li[2], Wenying Wang[1,*] and Wenlong Li[2,*]

[1] Department of Life Sciences, Qinghai Normal University, Xining 810008, P.R. China
[2] State Key Laboratory of Herbage Improvement and Grassland Agro-Ecosystems, College of Pastoral Agriculture Science and Technology, Lanzhou University, Lanzhou 730030, P.R. China

* Corresponding authors. Correspondence: wangwy0106@163.com (Wenying Wang).

Table S1 reports individual-algorithm and predictive ensemble evaluations. Table S2 compares random-partition and spatial block validation for all five species and seven algorithms.

Table S1 Model evaluation results

A Individual algorithms

Species	Algorithm	n	AUC (mean ± SD)	TSS (mean ± SD)
Stellera chamaejasme	GLM	25	0.946 ± 0.038	0.831 ± 0.063
S. chamaejasme	GBM	25	0.961 ± 0.011	0.841 ± 0.034
S. chamaejasme	CTA	25	0.907 ± 0.027	0.784 ± 0.044
S. chamaejasme	ANN	25	0.935 ± 0.021	0.822 ± 0.041
S. chamaejasme	SRE	25	0.778 ± 0.023	0.556 ± 0.046
S. chamaejasme	FDA	25	0.950 ± 0.016	0.809 ± 0.041
S. chamaejasme	RF	25	0.963 ± 0.011	0.838 ± 0.040
Thermopsis lanceolata	GLM	25	0.937 ± 0.013	0.758 ± 0.039
T. lanceolata	GBM	25	0.948 ± 0.011	0.788 ± 0.032
T. lanceolata	CTA	25	0.884 ± 0.031	0.718 ± 0.055
T. lanceolata	ANN	25	0.877 ± 0.033	0.677 ± 0.048
T. lanceolata	SRE	25	0.776 ± 0.032	0.552 ± 0.064
T. lanceolata	FDA	25	0.927 ± 0.019	0.736 ± 0.043
T. lanceolata	RF	25	0.953 ± 0.011	0.801 ± 0.035
Gymnaconitum gymnandrum	GLM	25	0.944 ± 0.020	0.793 ± 0.048
G. gymnandrum	GBM	25	0.952 ± 0.022	0.806 ± 0.053
G. gymnandrum	CTA	25	0.883 ± 0.042	0.730 ± 0.076
G. gymnandrum	ANN	25	0.905 ± 0.060	0.753 ± 0.106

Species	Algorithm	n	AUC (mean ± SD)	TSS (mean ± SD)
G. gymnandrum	SRE	25	0.783 ± 0.044	0.567 ± 0.088
G. gymnandrum	FDA	25	0.947 ± 0.019	0.791 ± 0.046
G. gymnandrum	RF	25	0.953 ± 0.022	0.818 ± 0.056
Oxytropis kansuensis	GLM	25	0.910 ± 0.028	0.738 ± 0.068
O. kansuensis	GBM	25	0.932 ± 0.029	0.770 ± 0.058
O. kansuensis	CTA	25	0.865 ± 0.040	0.708 ± 0.068
O. kansuensis	ANN	25	0.898 ± 0.042	0.708 ± 0.082
O. kansuensis	SRE	25	0.763 ± 0.045	0.526 ± 0.090
O. kansuensis	FDA	25	0.917 ± 0.027	0.742 ± 0.064
O. kansuensis	RF	25	0.936 ± 0.029	0.780 ± 0.057
Ligularia virgaurea	GLM	25	0.952 ± 0.026	0.849 ± 0.055
L. virgaurea	GBM	25	0.963 ± 0.021	0.860 ± 0.059
L. virgaurea	CTA	25	0.912 ± 0.035	0.801 ± 0.073
L. virgaurea	ANN	25	0.931 ± 0.031	0.819 ± 0.056
L. virgaurea	SRE	25	0.782 ± 0.044	0.563 ± 0.088
L. virgaurea	FDA	25	0.960 ± 0.020	0.851 ± 0.059
L. virgaurea	RF	25	0.965 ± 0.020	0.858 ± 0.062

Values are means ± sample SDs of Testing.data scores for RUN-labelled random-partition evaluations. Full-data fits were excluded, and all available evaluations were included without applying the ensemble-selection threshold. For every species, five pseudo-absence datasets and five random partitions per dataset yielded 25 evaluations per algorithm. n denotes evaluations, not occurrence records. ROC in the exported output is reported as AUC.

Table S1 Model evaluation results continued

B Predictive ensembles

Species	Ensemble	AUC	TSS	TSS cutoff (0–1 scale)
Stellera chamaejasme	EMmean	0.979	0.872	0.683
S. chamaejasme	EMca	0.985	0.880	0.828
S. chamaejasme	EMwmean	0.979	0.873	0.688
Thermopsis lanceolata	EMmean	0.975	0.830	0.620
T. lanceolata	EMca	0.980	0.847	0.384
T. lanceolata	EMwmean	0.976	0.832	0.584
Gymnaconitum gymnandrum	EMmean	0.976	0.835	0.407
G. gymnandrum	EMca	0.981	0.850	0.485
G. gymnandrum	EMwmean	0.976	0.835	0.414
Oxytropis kansuensis	EMmean	0.969	0.817	0.555
O. kansuensis	EMca	0.977	0.831	0.545
O. kansuensis	EMwmean	0.969	0.818	0.555
Ligularia virgaurea	EMmean	0.980	0.884	0.498
L. virgaurea	EMca	0.985	0.885	0.763
L. virgaurea	EMwmean	0.980	0.884	0.502

Ensemble values are single Testing.data scores exported for ensembles merging algorithms, runs and pseudo-absence datasets. They are not means ± SD or independent spatial hold-out scores and should not be directly interpreted as improvements over the individual-algorithm summaries in part A. Cutoffs were taken from the TSS rows of the ensemble evaluation output and divided by 1000. EMwmean supplied the reported habitat projections; EMmean and EMca are included for completeness. EMcv is a coefficient-of-variation output with NA evaluation entries and is not listed as a predictive ensemble.

Abbreviations: GLM, generalized linear model; GBM, generalized boosting model; CTA, classification tree analysis; ANN, artificial neural network; SRE, surface range envelope; FDA, flexible discriminant analysis; RF, random forest; AUC, area under the receiver operating characteristic curve; TSS, true skill statistic; SD, standard deviation. EMmean, unweighted mean ensemble; EMca, committee-averaging ensemble; EMwmean, TSS-weighted mean ensemble.

Table S2 Comparison of random and spatial validation

Species	Algorithm	Random AUC	Spatial AUC	Random TSS	Spatial TSS
Stellera chamaejasme	GLM	0.946 ± 0.038	0.887 ± 0.062	0.831 ± 0.063	0.770 ± 0.130
S. chamaejasme	GBM	0.961 ± 0.011	0.925 ± 0.036	0.841 ± 0.034	0.789 ± 0.113
S. chamaejasme	CTA	0.907 ± 0.027	0.837 ± 0.133	0.784 ± 0.044	0.674 ± 0.189
S. chamaejasme	ANN	0.935 ± 0.021	0.929 ± 0.054	0.822 ± 0.041	0.827 ± 0.080
S. chamaejasme	SRE	0.778 ± 0.023	0.747 ± 0.104	0.556 ± 0.046	0.493 ± 0.211
S. chamaejasme	FDA	0.950 ± 0.016	0.925 ± 0.032	0.809 ± 0.041	0.794 ± 0.108
S. chamaejasme	RF	0.963 ± 0.011	0.928 ± 0.028	0.838 ± 0.040	0.787 ± 0.075
Thermopsis lanceolata	GLM	0.937 ± 0.013	0.878 ± 0.062	0.758 ± 0.039	0.697 ± 0.135
T. lanceolata	GBM	0.948 ± 0.011	0.912 ± 0.040	0.788 ± 0.032	0.736 ± 0.130
T. lanceolata	CTA	0.884 ± 0.031	0.814 ± 0.128	0.718 ± 0.055	0.608 ± 0.188
T. lanceolata	ANN	0.877 ± 0.033	0.871 ± 0.063	0.677 ± 0.048	0.682 ± 0.096
T. lanceolata	SRE	0.776 ± 0.032	0.745 ± 0.126	0.552 ± 0.064	0.489 ± 0.252
T. lanceolata	FDA	0.927 ± 0.019	0.902 ± 0.039	0.736 ± 0.043	0.721 ± 0.124
T. lanceolata	RF	0.953 ± 0.011	0.918 ± 0.033	0.801 ± 0.035	0.750 ± 0.087
Gymnaconitum gymnandrum	GLM	0.944 ± 0.020	0.885 ± 0.066	0.793 ± 0.048	0.732 ± 0.144
G. gymnandrum	GBM	0.952 ± 0.022	0.916 ± 0.033	0.806 ± 0.053	0.754 ± 0.114
G. gymnandrum	CTA	0.883 ± 0.042	0.813 ± 0.109	0.730 ± 0.076	0.620 ± 0.163
G. gymnandrum	ANN	0.905 ± 0.060	0.899 ± 0.059	0.753 ± 0.106	0.758 ± 0.090
G. gymnandrum	SRE	0.783 ± 0.044	0.752 ± 0.105	0.567 ± 0.088	0.504 ± 0.204
G. gymnandrum	FDA	0.947 ± 0.019	0.922 ± 0.034	0.791 ± 0.046	0.776 ± 0.114
G. gymnandrum	RF	0.953 ± 0.022	0.918 ± 0.033	0.818 ± 0.056	0.767 ± 0.067
Oxytropis kansuensis	GLM	0.910 ± 0.028	0.851 ± 0.061	0.738 ± 0.068	0.677 ± 0.135
O. kansuensis	GBM	0.932 ± 0.029	0.895 ± 0.039	0.770 ± 0.058	0.718 ± 0.109
O. kansuensis	CTA	0.865 ± 0.040	0.795 ± 0.128	0.708 ± 0.068	0.598 ± 0.178
O. kansuensis	ANN	0.898 ± 0.042	0.891 ± 0.052	0.708 ± 0.082	0.713 ± 0.088
O. kansuensis	SRE	0.763 ± 0.045	0.732 ± 0.126	0.526 ± 0.090	0.463 ± 0.254
O. kansuensis	FDA	0.917 ± 0.027	0.892 ± 0.034	0.742 ± 0.064	0.727 ± 0.109
O. kansuensis	RF	0.936 ± 0.029	0.901 ± 0.028	0.780 ± 0.057	0.729 ± 0.077
Ligularia virgaurea	GLM	0.952 ± 0.026	0.893 ± 0.063	0.849 ± 0.055	0.788 ± 0.129

Species	Algorithm	Random AUC	Spatial AUC	Random TSS	Spatial TSS
L. virgaurea	GBM	0.963 ± 0.021	0.926 ± 0.030	0.860 ± 0.059	0.808 ± 0.107
L. virgaurea	CTA	0.912 ± 0.035	0.842 ± 0.119	0.801 ± 0.073	0.691 ± 0.171
L. virgaurea	ANN	0.931 ± 0.031	0.925 ± 0.057	0.819 ± 0.056	0.824 ± 0.091
L. virgaurea	SRE	0.782 ± 0.044	0.751 ± 0.120	0.563 ± 0.088	0.500 ± 0.234
L. virgaurea	FDA	0.960 ± 0.020	0.935 ± 0.032	0.851 ± 0.059	0.835 ± 0.110
L. virgaurea	RF	0.965 ± 0.020	0.930 ± 0.034	0.858 ± 0.062	0.806 ± 0.081

Values are means ± sample SDs. Random-partition summaries include 25 evaluations per algorithm for each species, based on five pseudo-absence datasets and five random partitions per dataset. Spatial summaries include five held-out folds per algorithm and species, using a single pseudo-absence dataset. Full-data fits were excluded from random-partition summaries, and no low-performing evaluations were excluded from either summary. ROC in the exported output is reported as AUC. The comparison is descriptive because the procedures differ in both data partitioning and pseudo-absence design; it is not a paired statistical test or an estimate of the isolated effect of spatial blocking. These results concern individual algorithms, not ensembles merging models across folds.

Abbreviations: GLM, generalized linear model; GBM, generalized boosting model; CTA, classification tree analysis; ANN, artificial neural network; SRE, surface range envelope; FDA, flexible discriminant analysis; RF, random forest; AUC, area under the receiver operating characteristic curve; TSS, true skill statistic; SD, standard deviation.
